# Mutant H3 histones drive human pre-leukemic hematopoietic stem cell expansion and promote leukemic aggressiveness

**DOI:** 10.1038/s41467-019-10705-z

**Published:** 2019-06-28

**Authors:** Meaghan Boileau, Margret Shirinian, Tenzin Gayden, Ashot S. Harutyunyan, Carol C. L. Chen, Leonie G. Mikael, Heather M. Duncan, Andrea L. Neumann, Patricia Arreba-Tutusaus, Nicolas De Jay, Michele Zeinieh, Katya Rossokhata, Yelu Zhang, Hamid Nikbakht, Carine Mouawad, Radwan Massoud, Felice Frey, Rihab Nasr, Jean El Cheikh, Marwan El Sabban, Claudia L. Kleinman, Rami Mahfouz, Mark D. Minden, Nada Jabado, Ali Bazarbachi, Kolja Eppert

**Affiliations:** 1 Division of Experimental Medicine, McGill University and McGill University Heath Centre Research Institute, Montreal, H4A 3J1 QC Canada; 2 Department of Experimental Pathology, Immunology, and Microbiology, American University of Beirut, Beirut, 1107 2020 Lebanon; 3 Department of Internal Medicine, American University of Beirut, Beirut, 1107 2020 Lebanon; 4 Department of Human Genetics, McGill University, Montreal, H3A 1B1 QC Canada; 5 Department of Pediatrics, McGill University and McGill University Heath Centre Research Institute, Montreal, H4A 3J1 QC Canada; 6 Research Institute of the McGill University Health Centre and McGill University, Montreal, H4A 3J1 QC Canada; 7 Lady Davis Institute for Medical Research, Jewish General Hospital, Montréal, H3T 1E2 QC Canada; 8 McGill University and Génome Québec Innovation Centre, Montreal, H3A 0G1 QC Canada; 9 Department of Anatomy, Cell Biology and Physiological Sciences, American University of Beirut, Beirut, 1107 2020 Lebanon; 10 Department of Pathology and Laboratory Medicine, American University of Beirut, Beirut, 1107 2020 Lebanon; 11 Princess Margaret Cancer Centre, University Health Network, University of Toronto, Toronto, M5G 2C1 ON Canada; 12 Department of Medicine, University of Toronto, Toronto, M5S 1A8 ON Canada

**Keywords:** Acute myeloid leukaemia, Cancer stem cells, Haematopoietic stem cells

## Abstract

Our ability to manage acute myeloid leukemia (AML) is limited by our incomplete understanding of the epigenetic disruption central to leukemogenesis, including improper histone methylation. Here we examine 16 histone H3 genes in 434 primary AML samples and identify Q69H, A26P, R2Q, R8H and K27M/I mutations (1.6%), with higher incidence in secondary AML (9%). These mutations occur in pre-leukemic hematopoietic stem cells (HSCs) and exist in the major leukemic clones in patients. They increase the frequency of functional HSCs, alter differentiation, and amplify leukemic aggressiveness. These effects are dependent on the specific mutation. H3K27 mutation increases the expression of genes involved in erythrocyte and myeloid differentiation with altered H3K27 tri-methylation and K27 acetylation. The functional impact of histone mutations is independent of *RUNX1* mutation, although they at times co-occur. This study establishes that H3 mutations are drivers of human pre-cancerous stem cell expansion and important early events in leukemogenesis.

## Abstract (web summary)

The role of histone mutations in leukemogenesis remains largely unexplored. In this study of AML, the authors show that histone mutations are early events that drive a more aggressive phenotype.

## Introduction

AML is a heterogeneous type of aggressive leukemia with poor survival, even with the use of intensive cytotoxic treatment^1,2^. It develops in a progressive manner as pre-leukemic cells accrue mutations leading to the development of full leukemic clones^3,4^. This can result in a mixed population of leukemic and related pre-leukemic clones in a patient with differing mutational composition^3,4^. Disrupted epigenetic control occurs during this process, and this is often driven by mutations in epigenetic regulators^5,6^. However, our incomplete understanding of what drives the altered epigenetic landscape in leukemia and when these factors contribute to disease development and progression impedes our ability to prevent or cure this disease.

Pre-cancerous expansions of mutated hematopoietic clones occur commonly in myeloid disease, such as AML, secondary AML (s-AML), and myelodysplastic syndrome (MDS), and rarely in healthy individuals. These pre-leukemic HSCs represent an intermediate step in leukemogenesis and put individuals at an increased risk for developing de novo or secondary hematological malignancies^3,4,7–10^. Mutations found in *ASXL1*, a regulator of histone methylation, are common alterations found in pre-leukemic HSCs^7,8,10^. Indeed, post-transcriptional modification of histones, including methylation and acetylation, is commonly deregulated in AML^6,11^. Mutations in genes associated with the polycomb repressive complex 2 (PRC2) that methylates histone H3 at K27, including *EZH2* and *ASXL1*, are involved in leukemogenesis^6,11^.

In addition to epigenetic regulators, mutations in histone genes have been previously identified in cancer. Mutations in H3 histone variants at K27 and G34 were first identified in pediatric brain tumors, followed by mutations in H3K36 in sarcomas^12–18^. The H3K27 mutations act dominantly to decrease global H3K27 tri-methylation (H3K27me3), at least partially due to inhibition of EZH2^19^. Examination of H3K27 mutations in hematological malignancies have identified them in T-acute lymphoid leukemia (H3.3), myelodysplastic syndrome (H3.3) and AML (H3.1)^20–22^. An initial study in AML suggested that histone mutations occur late in the leukemic progression and are present only in minor clones^22^. This is unexpected given the key roles of epigenetic modifiers in pre-leukemic development and histone mutations as founding mutations in pediatric brain tumors.

Here we use direct sequencing to examine all histone H3 variants in two cohorts of primary AML and remission samples and observe that histone mutations, including both novel and previously described mutations, can occur in pre-leukemic cells and contribute to the major leukemic clone. Comprehensive in vitro and in vivo analyses reveals that these oncohistones dramatically expand HSC populations and alter differentiation in the myeloid/erythroid lineage. In established human leukemia, H3K27 mutations increase proliferation and aggressive features. The H3 mutations occur at a higher frequency in s-AML. Our results demonstrate a role for oncohistones early in the leukemogenic process and in the expansion of human pre-cancerous stem cells.

## Results

### H3 histones are mutated in AML with a higher frequency in s-AML

To comprehensively examine the role of histone H3 in AML, we performed targeted MiSeq sequencing of the 16 histone H3 genes for 434 primary human AML samples from two cohorts (Supplementary Tables 1 and 2). We identified seven mutations in variants of histones H3.1 and H3.3 (see Table 1), including at amino acid K27; these were validated by Sanger sequencing (1.6%). The mutations are rare or not detectable in a large dataset of normal samples, suggesting that these are bona-fide somatic mutations in our leukemic samples (gnomAD: frequency of 0.0004% for Q69H, 0.04% for R8H, and not detected for the rest; number of alleles examined 144,258−276,692^23^). There is significant enrichment for s-AML in H3 mutant samples as three of the seven were found in s-AML (43%) compared to the general frequency of s-AML in our cohorts of 7.6% (Fisher’s exact test: *p* = 0.011; Tables 1 and 2). Overall, the incidence of all histone mutations in s-AML is 9%. Focusing on K27, where mutation has been shown to be embryonically lethal and confirmed as somatic in other cancers, we observed the same enrichment in s-AML for K27M and K27I (6%, Fisher’s exact test: *p* = 0.016)^22,24–26^.

**Table 1:** Histone mutations in 434 primary human AML samples by MiSeq or exome sequencing

Patient sample	Diagnosis	H3 histone (VAF)	Molecular profile (VAF)
064	AML secondary to myelofibrosis	HIST1H3F K27I (44.7%)	NPM1c FLT3 D469E (56.3%) ASXL1 L775X (42.5%) RUNX1-wt
105	De novo AML	HIST1H3H K27M (53.2%)	IDH2 R140Q (43.4%) DNMT3A R771X (45.3%) RUNX1 F389fs (45%) SRSF2 P95_R102del (52.3%)
105 Remission	Remission	HIST1H3H K27M (43.4%)	IDH2 R140Q (28%) DNMT3A R771X (38.7%) RUNX1 F389fs (38.7%) SRSF2 P95_R102del (37.2%)
083	De novo AML	HIST1H3A Q69H (42.2%)	t(9;11) RUNX1-wt
083 Remission	Remission	HIST1H3A Q69H (54.5%)	Negative RUNX1-wt
073	AML secondary to CMML	H3F3A A26P (50%)	Negative RUNX1-wt
8760	Secondary AML	H3F3A K27M (46.2%)	FLT3-TKD TET2 L615AfsX23 (55.60%) TET2 Y1579X (46.9%) SRSF2 P95H (47.5%) RUNX1-wt
0095	De novo AML	H3F3C R2Q (42%)	n.d. RUNX1-wt
0561	De novo AML	H3F3C R8H (99.9%)	NPM1c RUNX1-wt

**Table 2:** Clinical features of AML patients harboring a histone mutation

Patient sample	Age/sex	Diagnosis	WHO classification	ELN	Karyotype
064	59F	AML secondary to myelofibrosis	Myelodysplasia related changes	Favorable	48,XX,+8,+21
105	43M	De novo AML	Not otherwise specified	Adverse	46,XY
105 Remission	43M	Remission		Remission sample	46,XY
083	9F	De novo AML	Recurrent genetic abnormalities	Intermediate	46,XX,t(9;11)(p22;q23)
083 Remission	9F	Remission		Remission sample	46,XX
073	17F	AML secondary to CMML	With myelodysplasia related changes	Intermediate	49,XXX,+11,+19
8760	58M	Secondary AML	With myelodysplasia related changes	Intermediate	46,XY
0095	48F	De novo AML	Recurrent genetic abnormalities	Favorable	47,XX,der(3)t(3;16)(q25;q22),+8,der(16),inv(16)(p13.1q22),t(3;16)
561	53M	De novo AML	Not otherwise specified	Favorable	46,XY

### H3 mutations exist in pre-leukemic HSCs and major leukemic clones

To determine the clonal representation of histone mutations in AML, we performed exome sequencing of samples from patients 064, 105, and 8760 to identify co-occurring mutations, and we examined our MiSeq and exome data for variant allele frequency (VAF). The VAFs of the histone mutations were consistently high, establishing that the histone mutations were present in the major leukemic clone (Table 1). Furthermore, the high VAF indicated that the histone mutations potentially occurred in pre-leukemic founder clones in our samples. To address this, we examined remission samples from patients 105 and 083 for histone mutations (Table 1). The K27M mutation in the patient 105 diagnosis sample was present with a VAF of 43.4% in the patient’s remission sample. Likewise, in patient 083 the Q69H histone mutation from the diagnosis sample was also detected in the remission sample at a VAF of 54.5%. Further, the t(9;11) translocation present in the primary 083 sample was not detected in the remission sample, demonstrating the acquisition of the Q69H mutation prior to the t(9;11) translocation event. Our data establishes that histone mutations are present in the major leukemic clone in each patient and occur early in leukemogenesis in expanded, pre-leukemic HSCs.

### H3 mutations do not exclusively occur in RUNX1-mutated samples

To determine if histone mutations commonly co-occur with mutations in specific genes in our AML samples, we examined the exome sequencing data as well as MiSeq data on *IDH1, IDH2*, *ACVR1*, *BRAF*, and *PPM1D* (Table 1). In our data, histone mutations did not co-occur exclusively with specific other common mutations. Significantly, a recent study on H3K27 mutations in AML stated that these mutations occur exclusively in samples with *RUNX1* alterations, although the sample size was three AML samples^22^. We determined the *RUNX1* status in our samples and observed a *RUNX1* mutation in only one of the seven AML samples harboring a histone mutation (Table 1). More specifically, a *RUNX1* mutation co-occurred in only one of three K27 mutated samples. Thus, *RUNX1* mutations are not obligate co-factors for these histone mutations.

### Q69H, K27M, and K27I mutations alter differentiation in vitro

**Fig. 1:**
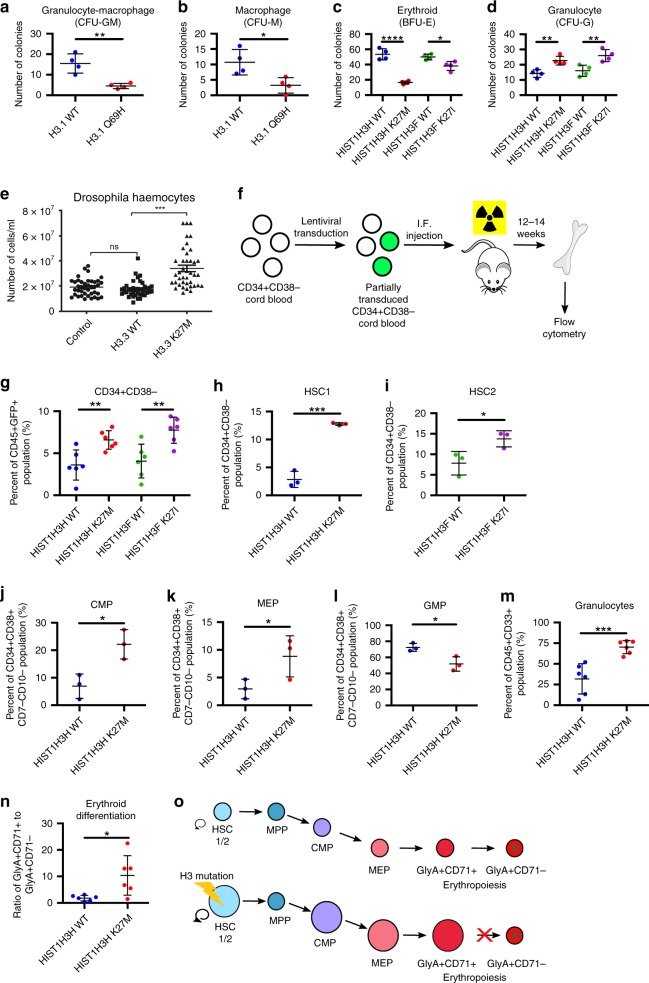
H3.1 mutations alter HSC frequency and hematopoietic differentiation in vitro and in vivo. Number of **a** granulocyte-macrophage colonies and **b** macrophage colonies (CFU-M) from CD34^+^ sorted human cord blood transduced with *HIST1H3H* WT or Q69H. **c**, **d** Number of (**c**) erythroid (BFU-E) and **d** granulocytic (CFU-G) colonies from CD34^+^ sorted human cord blood transduced with *HIST1H3H* WT/K27M or *HIST1H3F* WT/K27I. For **a**–**d** *n* = 4 and is representative of three independent experiments. **e** Circulating haemocytes in WT larvae (control), larvae expressing H3.3WT and H3.3K27M counted by Neubauer haemocytometer (*n* = 40). Data represent mean ± standard error of the mean. **f** Design of in vivo xenotransplantation of transduced CB cells injected into sublethally-irradiated NSG mice. **g**–**n** Flow cytometry analysis of populations from the bone marrow of the injected femur of mice xenotransplanted with CD34^+^CD38^-^ human cord blood cells transduced with *HIST1H3H* WT/K27M or *HIST1H3F* WT/K27I after 12–14 weeks. Data is representative of two independent experiments. **g** Frequency of CD34^+^CD38^-^ HSPCs in the CD45^+^GFP^+^ population (*n* = 6). **h** Frequency of HSC1 (CD45RA^−^CD90^+^CD49f^+^) and **i** HSC2 (CD45RA^-^CD90^−^CD49f^+^) in the CD34^+^CD38^−^ population. **j** Frequency of CMP (CD135^+^CD45RA^−^), **k** MEP (CD135^−^CD45RA^−^) and **l** GMP (CD135^+^CD45RA^+^) in the CD34^+^CD38^+^CD7^−^CD10^−^ population. Data for **h**–**l** represents pooled pairs of samples; *n* = 3. **m** Frequency of granulocytes (CD33^dim^, SSC^high^) in the CD45^+^ population and **n** ratio of CD71^+^ erythroid cells to CD71- erythroid cells in the CD45^−^GlyA^+^ populations (*n* = 6). **o** Schematic depicting the changes in frequency of HSCs and the block of differentiation in the erythroid lineage with H3.1 K27M/I mutations. See Supplementary Figs. 1 and 3 for gating strategy used. Data represents mean ± standard deviation. Statistical analysis was performed by two-way Student’s *t*-tests. *p ≤ 0.05, **p ≤ 0.01, ***p ≤ 0.001, ****p ≤ 0.0001

The presence of histone mutations in clonally expanded hematopoietic cells suggests that H3 mutations can alter normal hematopoietic stem or progenitor cell function. To examine this, we performed in vitro colony formation unit (CFU) assays in normal human CD34^+^ cord blood cells. The H3.1 Q69H mutation distorted differentiation and caused a decrease in myeloid colonies (CFU-GM and CFU-M; Fig. 1a, b). Previously, K27 mutations in H3.3 were shown to lead to an increase in CFU-G/GM colonies, although H3.1 mutations were not examined^22^. Using a complete cytokine cocktail allowing for detection of a wide range of human progenitor cells, we observed that human CD34^+^ cord blood cells transduced with H3.1 K27 mutants produce significantly fewer erythroid (BFU-E) and more granulocytic (CFU-G) colonies compared to control cells (Fig. 1c, d).

To confirm the fundamental impact of histone mutations on hematopoiesis, we examined them in a primitive blood system. *Drosophila melanogaster* that overexpress H3K27M mutated histones have increased circulating haemocytes in larvae compared to wildtype (Fig. 1e). This supports a critical role for histones in activating cellular innate immune response in *Drosophila* and reflects the central importance of the H3K27 histone mutations.

### H3.1 K27M/I drives HSC expansion and alters differentiation in vivo

Next, we assessed the functional effect of histone H3.1 mutations in HSCs, potentially leading to pre-leukemic expansion. A previous study showed that the expression of H3.3 K27 mutants in human CD34^+^ cells led to an expansion of phenotypically identified HSCs in vitro but a decrease in chimerism in vivo, suggesting that H3.3 K27 mutations could have an impact on HSC function despite the ambiguity of these results^22^. We performed quantitative in vivo functional HSC assays and used K27 mutants in H3.1 variants, as mutations in H3.3 are rarely found in AML. We transduced human CD34^+^CD38^−^ cells with lentiviral vectors to express HIST1H3H (K27M or wildtype) or HIST1H3F (K27I or wildtype) and transplanted these into mice (Fig. 1f). There was a substantial increase in the stem cell-enriched population (CD34^+^CD38^−^) in the mutant samples compared to controls after 14 weeks (Fig. 1g). Within this population, the K27M mutant drove a large expansion in the most primitive human HSC population (HSC1: CD45RA^−^CD90^+^CD49f^+^) while K27I expanded the HSC2 population (CD45RA^−^CD90^−^CD49f^+^; Fig. 1h, i, see Supplementary Fig. 1 for gating strategy and Supplementary Fig. 2 for complete data). To functionally assess these HSCs, we re-injected the transduced cells (CD45^+^GFP^+^) into secondary mice and measured engraftment after 14 weeks. We injected less than one million CD45^+^ cells, which is expected to contain few HSCs and does not generally result in robust engraftment in secondary mice. The mice injected with K27 mutant cells were highly engrafted (average 30% engraftment; 6/6 mice) while only one control mouse was engrafted (1/6 mice, 3.1% engraftment), confirming that K27 histone mutations expand functional HSCs in long-term assays (Mann–Whitney test: *p* = 0.004) (Table 3). Thus, histone mutations enlarge the mutant HSC population, consistent with the clonal expansion we observed in patient samples.

**Table 3:** Human CD45^+ ^engraftment of the right femur of NSG-S mice 14 weeks after secondary transplantation with CD45^+^GFP^+ ^human cord blood transduced with indicated genes

Sample	Cell number injected	Percent engrafted mice (>1%)	Engraftment (%)
HIST1H3H WT	975,000	0% (0/3)	0.07
	975,000		0.11
	975,000		0.09
HIST1H3H K27M	975,000	100% (3/3)	24.7
	975,000		30.9
	975,000		74.3
HIST1H3F WT	852,000	33% (1/3)	0
	852,000		3.1
	852,000		0.43
HIST1H3F K27I	852,000	100% (3/3)	24.70
	852,000		1.66
	852,000		22.80

We then examined whether the K27 histone mutations altered the in vivo differentiation of hematopoietic cells derived from the HSCs. After 14 weeks in vivo, we observed an expansion of common myeloid progenitors (CMPs) and megakaryocyte-erythroid progenitors (MEPs) and altered frequencies of multiple other populations in the K27M xenotransplant experiments, including granulocyte-monocyte progenitors (GMPs) (Fig. 1j–l and Supplementary Fig. 2 for complete analysis). The K27I mutation affected differentiation to a lesser extent than K27M in keeping with previous observations of the impact of the different amino acid substitutions on histone methylation (Supplementary Fig. 2)^19,27^. In the mature cells and lineage-committed fractions we detected an increase in granulocytes in the mutants (Fig. 1m, Supplementary Figs. 3 and 4). Moreover, we observed a blockage of differentiation in the erythroid lineage in the mutants with an increase in GlyA^+^CD71^+^ cells but a decrease in the downstream GlyA^+^CD71^–^ cells (Fig. 1n, Supplementary Fig. 4). This is consistent with the CFU results. Thus, hematopoietic cells with histone H3.1 mutants have altered myeloid differentiation with a bias towards the erythroid-megakaryocyte lineage and a blockage downstream in erythroid maturation (Fig. 1o).

### H3.1 K27M/I increase human AML proliferation and aggressiveness

**Fig. 2:**
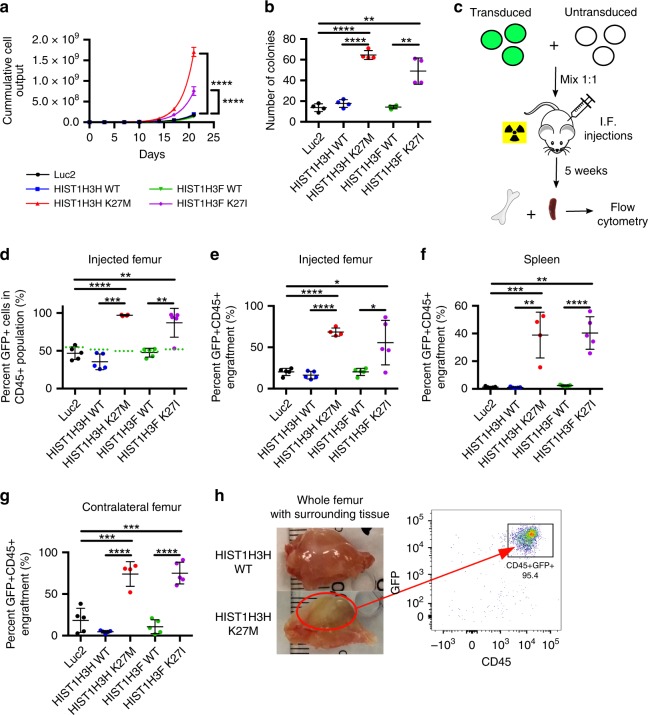
H3.1 K27M/I mutations increase the proliferation of AML cells in vitro and in vivo. **a** In vitro cell proliferation assay of TEX cells transduced with *HIST1H3H* WT (blue), *HIST1H3H* K27M (red), *HIST1H3F* WT (green), *HIST1H3F* K27I (pink), and Luc2 control (black) (*n* = 3, representative of two independent experiments). **b** Colony formation unit assay of TEX cells transduced with *HIST1H3H* WT, *HIST1H3H* K27M, *HIST1H3F* WT, *HIST1H3F* K27I, and Luc2 control (*n* = 4, representative of three independent experiments). **c** Design of in vivo xenotransplantation of transduced TEX cells mixed equally with untransduced TEX cells into sublethally-irradiated NSG-S mice. **d**–**g** Flow cytometry analysis of mice xenotransplanted with TEX cells transduced with *HIST1H3H* WT/K27M, *HIST1H3F* WT/K27I, or Luc2 control for 5 weeks. Data is representative of two independent experiments. **d** Percent GFP^+^ transduced cells in the human (CD45^+^) population 5 weeks post-injection of TEX cells. **e**–**g** Percent GFP^+^CD45^+^ engraftment of **e** the injected femur, **f** the spleen, and **g** the contralateral femur 5 weeks post-injection of TEX cells. **h** Representative image of injected femur and surrounding tissue of mice intrafemorally injected with TEX cells overexpressing HIST1H3H WT (top panel) or HIST1H3H K27M (bottom panel). Flow cytometry analysis indicates the solid mass consists of TEX cells (95.4% human CD45^+^ transduced cells) (*n* = 5). Data represents mean ± standard deviation. Statistical analysis was performed by two-way Student’s *t*-tests. *p ≤ 0.05, **p ≤ 0.01, ***p ≤ 0.001, ****p ≤ 0.0001

Next, to examine the role of oncohistones in established human AML, we examined TEX cells, a line derived from CD34^+^-enriched human cells transduced with the TLS-ERG oncogene, a fusion gene that occurs in s-AML/MDS^28^. Transduction with K27M/I mutant H3.1 histones led to a dramatic increase in proliferation compared to controls (Fig. 2a). In addition, these cells showed a 3-fold increase in functional stem/progenitor cells as determined by colony formation assays (Fig. 2b). To assess the impact of oncohistones in vivo, we injected a 1:1 mix of transduced and untransduced cells into the right femur of NSG-S mice and examined the bone marrow and spleens 5 weeks later (Fig. 2c). The TEX cells transduced with mutant histones outcompeted untransduced TEX cells with a 9:1 ratio of transduced to untransduced after 5 weeks while wildtype or Luc2-control transduced cells did not show this competitive advantage (Fig. 2d). Additionally, TEX cells transduced with wildtype histones or Luc2-control displayed an average engraftment of 21% in the injected femur while mutant TEX cells engrafted at >60%, indicating that mutant TEX cells are more successful in outcompeting endogenous mouse bone marrow cells compared to controls (Fig. 2e). Furthermore, mutant TEX cells presented with additional features of a more aggressive disease. They gained the ability to engraft the spleen (Fig. 2f) and had dramatically increased engraftment in the contralateral femur (Fig. 2g). Additionally, only TEX cells transduced with K27M/I mutants, but not wildtype histones or Luc2-control, produced solid masses of leukemic cells at the site of injection (Fig. 2h). Overall, we observed that H3.1 K27M/I mutant histones increase the proliferation and aggressiveness of human leukemic cells.

### H3.1 K27M/I alter gene expression, H3K27 methylation and acetylation

**Fig. 3:**
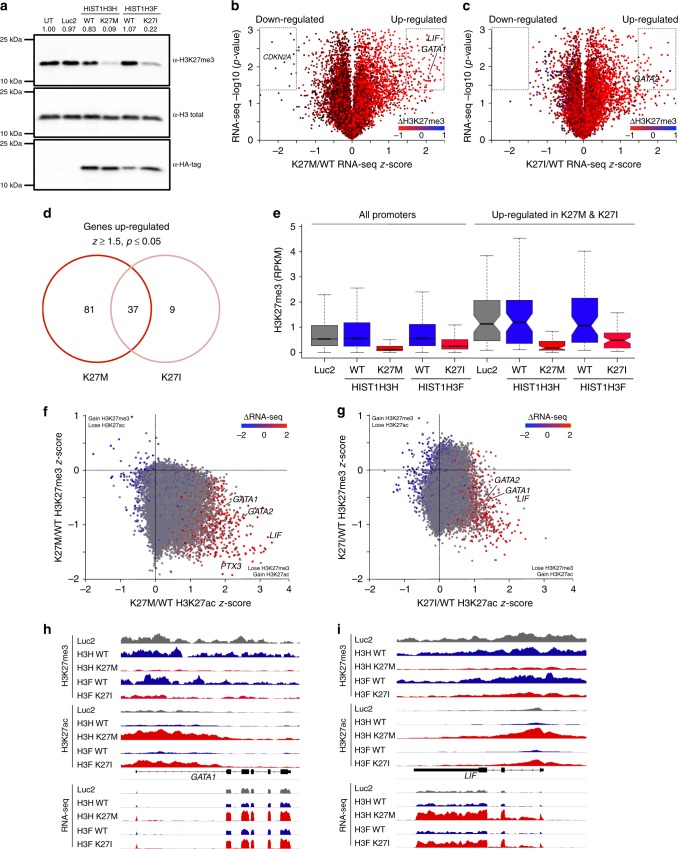
H3.1 K27M/I mutations modify H3K27me3 and H3K27ac marks and alter gene expression in leukemic cells. **a** Western blot analysis of H3K27me3 levels from total histone protein from TEX cells transduced with *HIST1H3H* WT/K27M, *HIST1H3F* WT/K27I, Luc2 control, or untransduced (UT) cells. **b** Volcano plot depicting genes showing differential expression in *HIST1H3H* K27M relative to WT. H3K27me3 change of K27M/WT is overlaid as a heatmap, with red and blue representing loss and gain, respectively. Dashed gates indicate genes called as being significantly down- or upregulated, using threshold of |*z*-score| >1.5, *p*-value <0.05. **c** Volcano plot depicting genes showing differential expression in *HIST1H3F* K27I relative to WT, as in **b**. **d** Venn diagram showing overlap of significantly upregulated genes observed in K27M and K27I expressing TEX cells. **e** Box-whisker plot showing change of promoter-specific H3K27me3 in the TEX cells, comparing all annotated promoters and the subset that showed up-regulation in both K27M and K27I. The lower and upper whisker represents the minimum and maximum, respectively, after removing outliers where the upper whisker = min(max(x), Q3+1.5 × IQR) and lower whisker = max(min(x), Q1−1.5 × IQR), where IQR = Q3-Q1. The two ‘hinges’ are versions of the first and third quartile. The notches extend to ± 1.58 IQR/sqrt(n) representing a confidence interval. IQR stands for interquartile range. Center line indicates median. **f**, **g** two-dimensional scatterplot depicting the change of H3K27me3 and H3K27ac at all annotated promoters in **f** K27M and **g** K27I overexpressing TEX cells. Corresponding K27M/WT RNA-seq *z*-score is overlaid as a heatmap, with red and blue representing up- and down-regulation, respectively. **h**, **i** Genome browser snapshot of the **h** GATA1 locus and **i** *LIF* locus with RNA-seq and Rx-normalized H3K27me3 and H3K27ac data

We next examined the underlying effects of histone mutations on H3K27me3, H3K27 acetylation (H3K27ac), and gene expression. Immunoblotting revealed that TEX cells expressing the K27 mutated H3.1 histones lead to a global decrease in H3K27me3 compared to controls (Fig. 3a), as previously observed^22,29^. RNA-seq analysis of TEX cells revealed that K27 mutations alter gene expression (Fig. 3b, c; see Supplementary Data 1 for complete RNA-seq and ChIP-seq data). Most of the affected genes showed increased expression in both K27M and K27I cells (Fig. 3b, c). Specifically, we identified 118 genes as significantly upregulated in H3.1 K27M cells compared to only 46 genes in K27I cells (Fig. 3b–d, Supplementary Data 2 for gene lists). ChIP-seq analysis of all annotated promoters revealed a global decrease in H3K27me3 and an inverse correlation with gene expression (Fig. 3b, c, e). Consistent with transcriptome analysis, loss of H3K27me3 was more severe in TEX cells overexpressing K27M compared to the K27I mutation. We specifically examined the H3K27me3 levels at the promoters of the 118 K27M and 46 K27I upregulated genes. In wildtype cells, H3K27me3 enrichment levels at these promoters were higher than the median global promoter levels, while the K27 mutants both showed significant large decreases (Fig. 3d, e, h, i). Finally, we confirmed that the mutant and wildtype histones have a similar pattern of DNA localization in leukemic cells, indicating that the effects we observed are not driven by mutant-specific histone localization (Supplementary Fig. 5 and Supplementary Data 3). These analyses suggest that upregulated genes are direct targets of H3K27me3-mediated repression in wildtype cells, and upon expression of K27M, and to a lesser extent K27I, the reduction in H3K27me3 leads to up-regulation.

Next, we examined if these upregulated genes may account for the functional differences seen in K27 mutant cells compared to wildtype cells. Ontology analysis revealed processes involved in hematopoietic differentiation, including general myeloid and erythroid differentiation (Table 4). In order to capture a broad range of differentially expressed genes, we performed a second analysis using a more generous threshold of detection. We identified 360 upregulated and 78 downregulated genes for K27M and 190 upregulated and 52 downregulated genes for K27I (Supplementary Fig 6, Supplementary Data 2). Additional processes identified include immune regulation, chemotaxis and adhesion, with interferon gamma mediated signaling identified specifically in the downregulated genes (Supplementary Fig 6e).

**Table 4:** Gene ontology enrichment analysis of significantly upregulated genes in cells overexpressing K27M compared to WT

GO biological process (genes upregulated in K27M)	Hits^a^	FDR^b^ *p*-value	Genes
Leukotriene production involved in inflammatory response (GO:0002540)	2/2	0.0395	*ALOX5, ALOX5AP*
Interleukin-5-mediated signaling pathway (GO:0038043)	2/2	0.0387	*IL5RA, CSF2RB*
Eosinophil fate commitment (GO:0035854)	2/2	0.0383	*GATA1, GATA2*
Regulation of primitive erythrocyte differentiation (GO:0010725)	2/2	0.0379	*GATA1, GATA2*
Negative regulation of interleukin-10 production (GO:0032693)	3/17	0.0415	*FCGR2B, EPX, PRG2*
Positive regulation of Ras protein signal transduction (GO:0046579)	5/52	0.00915	*P2RY10, CSF, GPR65, NTRK1, ALS2*
Actin cytoskeleton reorganization (GO:0031532)	5/58	0.0137	*ANXA1, GPR65, S1PR1, FER, FRY*
JAK-STAT cascade (GO:0007259)	4/47	0.0454	*CLC, LIF, PKD2, FER*
Positive regulation of myeloid cell differentiation (GO:0045639)	5/85	0.0431	*SCIN, CSF1, LIF, GATA2, GATA1*
Regulation of gliogenesis (GO:0014013)	6/104	0.0182	*CLC, SLC45A3, CSF1, LIF, CXCR4, PTX3*

^a^Hits represent the total number of genes identified over the total number of genes annotated for the specific GO term.

^b^FDR: Fisher’s exact test, corrected for multiple testing

It has been demonstrated that K27 mutations can alter the distribution of the H3K27ac activation mark^19,30^. Intersection of H3K27ac ChIP-seq results with RNA-seq and H3K27me3 in TEX cells showed that loss of H3K27me3 was not concomitant with H3K27ac gain *per se*. Rather, the promoters of upregulated genes, such as *GATA1* and *LIF*, have higher H3K27ac (Fig. 3f–i). Thus, global loss of the repressive H3K27me3 in H3.1 K27 mutants results in ectopic gain of H3K27ac and up-regulation of hematopoietic genes.

One of the few genes whose expression was significantly decreased in TEX cells transduced with K27 mutant H3.1 histones is *CDKN2A*, suggesting that histone mutations drive proliferation through cell division (Supplementary Data 1). Suppressed expression of *CDKN2A* has been observed in glioma cells with H3K27 mutations and rescued with EZH2 inhibitors^31,32^. However, we observed a protective role for the H3.1 K27 mutations against treatment with the EZH2 inhibitor UNC1999 (Supplementary Fig. 7). In addition, we show that H3.1 K27M AML cells are not more sensitive to drugs targeting other epigenetic regulators such as inhibitors against the histone demethylase JMJD3 (GSK-J4), BRD4 (JQ1) and histone deacetylases (vorinostat, trichostatin A and panobinostat) (Supplementary Fig. 7). Overall, the functional alterations driven by H3.1 K27M epigenetic modifications are robust and difficult to treat with such therapies.

## Discussion

An early step in the leukemogenic process is the alteration of epigenetic regulators, resulting in a disrupted epigenetic landscape in leukemic cells. The mutations involved and their effects are complex and not fully understood. The well-established role of histone modifications in leukemia led us to examine two cohorts of AML samples in which we identified mutations in histone H3 variants. These mutations expand functional HSCs (K27M/I), alter differentiation (K27M/I and Q69H), and lead to an increase in proliferation and other indicators of aggressiveness in leukemia (K27M/I). This represents a fundamental new mechanism for the epigenetic alterations driving leukemogenesis.

We observed a higher frequency of H3 mutations in s-AML. Mutations involved with PRCs such as *ASXL1, EZH2*, and *BCOR* are linked to s-AMLs and their antecedent MDS^33–35^. A mutation in these genes is over 95% specific for s-AML^33^. Thus, the enrichment of histone mutations in s-AML in our cohorts is consistent with the altered regulation of histone methylation seen in s-AML/MDS. In further support of a role for histone mutations in s-AML and prior MDS, an H3F3A K27N mutation with unknown function has been previously observed in an MDS sample^21^.

Substantial clonal expansion of the normal bone marrow, resulting in high VAFs, has been observed in AML patients, and in s-AML patients in particular^5,36,37^. High VAFs have been found in leukemic samples but also VAFs of approximately 50% have been observed for known oncogenic driver mutations in bone marrow cells in remission samples. In the case of MDS and s-AML nearly all the bone marrow cells are clonal, even though by definition malignant cells are fewer than 20% in MDS patients^36^. Thus, histone mutations may act as other oncogenic drivers in myeloid malignancies and contribute to the almost complete clonal dominance of pre-leukemic cells over wildtype cells in the bone marrow.

Unlike a recent study, we did not find that the impact of histone mutations is reliant on mutated *RUNX1*^22^. Only one of seven AML samples with a histone mutation in our cohorts had a corresponding *RUNX1* alteration. Furthermore, histone mutations trigger substantial functional changes in leukemic TEX cells and normal cord blood cells, both of which are wildtype for *RUNX1* and the *AML1/ETO* fusion. As discussed above, s-AML represents a subtype of AML with specific associated mutations^33^. This includes *RUNX1* and mutations in this gene have been shown to co-occur with mutations in *ASXL1* and *EZH2*^33,38,39^. The high frequency of *RUNX1* mutations in s-AML may account for the previous observation of co-occurrence of histone H3K27 mutations with *RUNX1* alterations^22^.

In one sample in our cohort we observed both an *ASXL1* and *HIST1H3F* K27I mutation. However, *ASXL1* mutations can occur with *EZH2* mutations in myeloid malignancies^33,40,41^, suggesting that *ASXL1* mutations do not phenocopy histone H3.1 or *EZH2* mutations. This highlights the complexity of epigenome regulation in cancer as the co-occurrence of mutations in genes within shared pathways indicates that they have unique roles.

Histone mutations may serve as potential therapeutic targets in AML patients, either by treating leukemic cells or pre-leukemic clones. Gliomas harboring H3K27M mutations have been shown to be more sensitive to EZH2 inhibitors than wildtype H3 gliomas. This effect is thought to be mediated by the de-repression of *CDKN2A*^31^. Despite also seeing a decrease in *CDKN2A* expression in AML cells expressing H3.1 K27M/I, we did not observe increased sensitivity to EZH2 inhibition. In addition, mutant cells are not more sensitive to drugs targeting components of additional epigenetic processes such as JMDJ3, BRD4 and HDACs. Therefore, these compounds may not be appropriate for H3K27 mutant myeloid malignancies and other therapeutics that can modulate the epigenome may be necessary.

Overall, our data establishes that histone mutations can drive pre-leukemic HSC expansion, with concurrent altered myeloid/erythroid lineage differentiation, and contribute to leukemic aggressiveness. Our data is evidence of an oncohistone-driven expansion of pre-neoplastic primary human stem cells and we speculate that this occurs in cancer types beyond leukemia.

## Methods

### Sample characteristics and pathological review

Two cohorts were included in the study. The first consisted of 122 patients diagnosed with AML at the American University of Beirut Medical Center (AUBMC), and the second one included 312 patients diagnosed with AML at the University Health Network, Toronto. The study was approved by the institutional review board at both centers (UHN; REB# 01-0573-C), and a shared database with strictly defined variables was created to avoid bias. All subjects involved in the study provided written, informed consent.

Bone marrow samples at AUBMC were obtained from AML patients who had their DNA previously collected for routine diagnostic purposes and stored in the Department of Pathology and Laboratory Medicine as part of the College of American pathologist accreditation requirements from January 2005 to January 2015. Karyotypic analysis (Supplementary Table 1) “Other” category includes+8, −7, -Y,+11,+13,+15,+19,+21,+22, and alterations such as rare translocations and rare deletions.

### MiSeq sequencing

We performed deep targeted sequencing on DNA from 442 AML samples from Lebanon (*N* = 122) and Toronto (*N* = 312) using an Illumina MiSeq platform (McGill University). The MiSeq panel covers exon 2 of H3.3 (3 H3.3 genes), coding regions of H3.1 and H3.2 isoforms (10 H3.1 and 3 H3.2 genes) as well as mutation hotspots in *IDH1* (codon 132), *IDH2* (codons 140 and 172), *ACVR1* (exons 6–9), *BRAF* (V600E) and *PPM1D* (exon 6). The sequencing data were analyzed as previously described^13,42^. We achieved median coverage of 7000 and 16,000 reads per region in the Lebanese and Toronto cohorts, respectively. Only those variants with allele frequency ≥10% and that were supported by at least 20 alternate reads were retained. These variants were then filtered for common SNPs found in public databases such as the 1000 Genomes Project, dbSNP, and the NHLBI Exome Variant Server (ESP). Sanger sequencing was performed on the *DNMT3A* gene with the full coding regions of the gene being sequenced in the 122 AML samples from Lebanon.

### Whole exome sequencing

Exomes were captured using the Agilent SureSelect All Exon kit v5 kit according to the manufacturer’s instructions and described previously^24^. Exome sequencing was performed on samples from patients 064, 105, and 8760, including the remission sample for 105. Exome sequencing was performed on samples identified with a H3K27 mutation to identify any co-occurring mutations. The enriched libraries were sequenced on either the Illumina HiSeq 2500 or HiSeq 4000 with 100 bp paired-end reads. Sequence reads were mapped to the human reference genome (hg19) with Burrows-Wheeler Aligner (BWA)^43^, and duplicate reads were flagged using Picard (http://picard.sourceforge.net) and excluded from further analyses. Variants were called using three different variant callers including SAMtools Mpileup^44^, FreeBayes version v1.1.0-4-gb6041c6 (Haplotype-based variant detection from short-read sequencing. E Garrison, G Marth - arXiv:1207.3907, 2012), and GATK haplotype caller version 3.8^45^, and were filtered to require at least 20% of reads supporting the variant call. In order to keep the high confidence variant calls, we only kept those variants that were called by at least two of three variant callers. Mutations were annotated using both ANNOVAR^46^ and custom scripts. Annotated variants were filtered against the common germline polymorphisms present in dbSNP135, the 1000 Genomes project^47^, NHLBI GO Exomes and an in-house database of approximately 3000 exomes previously sequenced at our center.

### Sanger sequencing of *RUNX1*

We performed Sanger sequencing of exons 3–8 of the *RUNX1* gene in AML samples from patients 073, 083, 095, 0561 and the remission sample from 083. The primer sequences are as follows:

RUNX1 exon 3 forward; GCTGTTTGCAGGGTCCTAA, reverse; CCTGTCCTCCCACCACCCTC. RUNX1 exon 4 forward; CATTGCTATTCCTCTGCAACC, reverse; TGCCATGAAACGTGTTTCAAGC.

RUNX1 exon 5 forward; TCAGGCCACCAACCTCATTCTG, reverse; CCAGCCCCAAGTGGATGCAC. RUNX1 exon 6 forward; AGCCCCAGTTTTAGGAAATCCAC, reverse; AGCATCAAGGGGAAACCCC. RUNX1 exon 7 forward; CCCACCCCACTTTACATATAATTG, reverse; CCAGCTCAGCTGCAAAGAATGTG. RUNX1 exon 8 forward; CCGCAACCTCCTACTCACTT, reverse; GCTTGTCGCGAACAGGAG^48^.

### Collection of cord blood and cell culture

Human cord blood was obtained from full-term deliveries from healthy donors according to the procedures approved by the Institutional Review Boards of Héma-Québec, Sainte-Justine’s Hospital, University of Montreal and the McGill University Health Centre. Written informed consent was given by women during pregnancy within the province of Québec. Mononuclear cells were obtained by centrifugation on Ficoll (GE Healthcare) and were enriched for CD34^+ ^cells using the EasySep CD34^+ ^positive selection kit (STEMCELL Technologies) as per the manufacturer’s protocol. CD34^+ ^enriched human cord blood was cultured in StemSpan SFEM II (STEMCELL Technologies) containing penicillin/streptomycin (Life Technologies), 10 ng/mL IL-6, 100 ng/mL SCF, 100 ng/mL FLT3L, 10ng/mL G-CSF, and 15 ng/mL TPO (Life Technologies).

TEX human leukemia cells (a gift from Dr. J. Dick, Toronto, Canada) were cultured in IMDM (Hyclone) containing 4mM l-glutamine (Life Technologies), 15% fetal bovine serum (FBS) (Wisent), penicillin/streptomycin (Life Technologies), 20 ng/ml SCF and 2 ng/mL IL-3 (Life Technologies). 293FT cells were cultured in high-glucose DMEM (Life Technologies) containing 10% Cosmic Calf Serum (CCS) (GE Healthcare Life Sciences), 0.1 mM MEM non-essential amino acids (Wisent), 6mM L-glutamine, 1Mm MEM sodium pyruvate (Life Technologies), penicillin/streptomycin and 500 μg/mL G-418 (Wisent). Cells were incubated at 37 °C with 5% CO_2_. Cells were routinely checked for mycoplasma contamination using the Mycoalert Detection kit (Lonza) and were negative.

### Lentiviral vector production

The previously described pSMAL vector modified from the MA1 lentiviral vector to have a Gateway cassette and SFFV promoter was used as the backbone for all generated lentiviral vectors^49,50^. *HIST1H3H* WT/K27M and *HIST1H3F* WT/K27I with a C-terminal HA-tag and attB sites for Gateway cloning were generated using GeneArt™ DNA Strings Fragments (Life Technologies). Luciferase modified for human expression (Luc2) was amplified out of the pGL4.51[luc2/CMV/NEO] vector (Promega). Genes were cloned into the pDONR221 vector (Invitrogen) using BP Clonase (Invitrogen) and subsequently cloned into the pSMAL vector using LR Clonase (Invitrogen) as per the manufacturer’s instructions. Lentiviral particles were produced in 293FT cells (Life Technologies) as previously. Viral titer was determined by adding serial dilutions of the concentrated viral particles onto 8227 AML cells and the percentage of GFP^+ ^cells was measured 96 h later by flow cytometry.

### Lentivirus transduction

pSMAL lentiviral vectors were used to transduce TEX and cord blood cells at an MOI of 3–5^49,51^. After 16 h, additional appropriate media was added and transduction efficiency was measured using flow cytometry for GFP after 96 h. In transduced TEX cells the K27I mutation accounts for 26% of the *HIST1H3F* reads and 4% of the total H3 reads with the K27M mutation at 20% of the *HIST1H3H* reads and 3.6% of the total H3 expression.

### Fluorescence-activated cell sorting and flow cytometry

To isolate transduced cells and identify specific populations of cord blood, cells were stained with desired surface markers in PBS with 2% cosmic calf serum (CCS) (Hyclone) and sorted using a BD FACSAria. Flow cytometry was performed using a BD LSRFortessa. Antibodies used are described below and in the supplementary methods. All antibodies were obtained from Biolegend and BD Biosciences. SYTOX Blue (Life Technologies) was used to distinguish live cells from dead cells.

### Antibody details

CD34 (APC, APC-Cy7; clone 581), CD38 (PE; clone HB-7), CD45 (AlexaFluor 700; clone 2D1), CD7 (Pe-Cy7; clone CD7-6B7), CD10 (Pe-Cy5; clone HI10a), CD45RA (BV650; clone HI100), FLT3 (PerCP-Cy5.5; clone BV10A4H2), CD90 (BV605; clone 5E10), CD49f (APC; clone GoH3), CD235a (PerCP-Cy5.5; clone HI264), CD71 (PE; clone MA712), CD41 (Pe-Cy7; clone HIP8), CD14 (PE-Dazzle; clone HCD14), CD33 (APC; clone WM53), CD3 (BV605; clone SK7), CD19 (PerCP-Cy5.5; clone HIB19), and CD56 (PE-Cy5; clone hCD56). All antibodies were obtained from Biolegend and BD Biosciences.

### Growth and methylcellulose colony-forming unit assays

Transduced TEX cells were cultured in the conditions described in the supplementary methods and harvested every 3 days, counted and reseeded. Cumulative growth was calculated over 21 days. Details for measuring viability after drug exposure are defined in the supplementary methods. Enriched methylcellulose (H4435 STEMCELL Technologies) was used for all assays. 2,000 TEX cells were plated into methylcellulose and colonies were counted 10 days later. 500 transduced cord blood cells sorted for GFP^+^CD34^+^ were plated into methylcellulose and colonies were classified and counted 14 days later. Colony types were identified by morphology and color, as per standard criteria.

### Drosophila stocks and generation of H3.3 WT and H3.3 K27M transgenic flies

Transgenic flies harboring human H3.3 WT and K27M histones were generated using the Phi C31 integrase system. A pUASg.attB Gateway vector containing the human cDNA with 1xHA (C-terminus) was injected into the ZH-attP line on the third chromosome zh-86Fb (3R 86F) for GAL4-UAS expression. A hemocyte-specific driver (Cg-GAL4.A) (#7011) was obtained from BDSC^52^. Flies were crossed at 25 °C. White W1118 flies were used as a wildtype control.

### Haemocyte counting

H3.3 WT and K27M flies were crossed to the Cg-Gal4 haemocyte driver. Third instar larvae of the indicated genotypes were bled into PBS (SIGMA) and haemocytes were counted. Forty larvae of each genotype (three independent crosses) were counted in each experiment and statistical analysis was performed using Student’s *t*-tests *P* < 0.05 (*n* = 40).

### Xenotransplantations

Mouse xenografts were performed according to protocols approved by McGill University and its affiliated Hospitals’ Research Institutes.

### TEX cells xenotransplant

NOD-scid IL2Rg^null^-3/GM/SF (NSG-S) mice were irradiated with 2.1 Gy using a X-RAD SmART Irradiator (Precision X-Ray, Inc) 24 h prior to intrafemoral injection of 2 million TEX cells (1:1 mixture of untransduced to transduced). Mice were sacrificed 5 weeks after injection (onset of illness) and cells from the injected femur, contralateral femur and spleen were collected for flow cytometry. Human engraftment (CD45^+^) and GFP percentage were evaluated by flow cytometry for all collected tissue.

### Cord blood xenotransplant

CD34^+^ enriched cord blood was fluorescence-activated sorted for CD34^+^CD38^−^. 10,000–20,000 cells per mouse were transduced with lentiviral vectors (MOI 3–5) and maintained in culture for 4 days. NOD-scid IL2Rgamma^null^ (NSG) mice were irradiated with 2.1 Gy 24 h prior and injected intrafemorally with the transduced cord blood cells. Mice were sacrificed 12–14 weeks after injection and bone marrow from the injected femur, contralateral femur and spleen were collected for flow cytometry analysis. Flow cytometry was done to evaluate human engraftment (human-specific CD45 and GFP) and hematopoietic differentiation (CD34, CD38, CD235a (GlyA), CD33, CD19, CD56, CD3, CD41, CD71, and CD14). Bone marrow cells from the injected femur were mouse cell-depleted (STEMCELL Technologies 19849) and lineage-depleted using EasySep^TM^ (STEMCELL Technologies 19356) as per the manufacturer’s protocol prior to analysis of primitive populations by flow cytometry using antibodies against CD34, CD38, CD7, CD10, CD45RA, FLT3, CD90, and CD49f.

### Secondary transplantation

GFP^+^CD45^+^ cells were sorted from pooled bone marrow from the right and left femurs of primary mice. 852,500 to 975,000 cells were injected intrafemorally into sublethally-irradiated NSG mice. Mice were sacrificed 14 weeks after injection and bone marrow from the injected femur, contralateral femur and spleen were collected for flow cytometry analysis. Flow cytometry was done to evaluate human engraftment (hCD45 and GFP).

### Immunoblotting

Total histones were extracted, run on SDS-PAGE and immunoblotted by conventional methods and described elsewhere^24^. Antibodies used were anti-H3K27me3 (1:2500; Millipore 07–449), anti-Total H3 (1:5000; ab1791), anti-HA (1:2500; CST 3724), and HRP-conjugated anti-rabbit IgG secondary antibody (1:5000, NA934V). Quantification of the ratios of H3K27me3/H3 total were calculated using the Image Lab 5.2.1 software. Uncropped immunoblots can be found in the Source Data.

### ChIP and RNA-sequencing on TEX cells

ChIP-seq and RNA-seq preparation and sequencing were carried out as described previously^24^. Cells were fixed with 1% formaldehyde (Sigma). Fixed cell preparations were washed, pelleted and stored at −80 °C. Sonication of lysed nuclei (lysed in a buffer containing 1% SDS) was performed on a BioRuptor UCD-300 for 60 cycles (10 s on 20 s off), centrifuged every 15 cycles, and chilled by 4 °C water cooler. Samples were checked for sonication efficiency using the criteria of 150–500 bp by gel electrophoresis. After sonication, the chromatin was diluted to reduce SDS level to 0.1% and before the ChIP reaction 2% of sonicated *Drosophila* S2 cell chromatin was spiked-in the samples for quantification of total levels of histone mark after the sequencing.

ChIP reaction was performed on a Diagenode SX-8G IP-Star Compact using Diagenode automated Ideal ChIP-seq Kit. For H3K27me3 and HA-Tag, 80 μl magnetic beads (Dynabeads M-280 Sheep Anti-Mouse IgG) were washed and then incubated with 2 μg of H3K27me3 antibody (Active Motif, #61017) and 2 million cells of sonicated cell lysate combined with protease inhibitors for 10 h, followed by 20 min wash cycle with provided wash buffers. For H3K27ac, 30 μl magnetic beads (Dynabeads Protein A) were washed and then incubated with 5 μg of H3K27ac antibody (Diagenode, C15410196). For HA-tag, the HA-tag antibody (Cell Signaling Technologies, 3724, rabbit monoclonal) was used at a 1:100 dilution. Reverse cross linking took place on a heat block at 65 °C for 4 h. ChIP samples were then treated with 2 μl RNase Cocktail at 65 °C for 30 min followed by 2 μl Proteinase K at 65 °C for 30 min. Then, samples were purified with QIAGEN MiniElute PCR purification kit as per manufacturers’ protocol. In parallel, input samples were reverse crosslinked and DNA was isolated following the same protocol. Library preparation was carried out using Kapa HTP Illumina library preparation reagents. In brief, 25 μl of ChIP sample was incubated with 45 μl end repair mix at 20 °C for 30 min followed by Ampure XP bead purification. A tailing: bead-bound sample was incubated with 50 μl buffer enzyme mix for 30 min at 30 °C, followed by PEG/NaCl purification. Adapter ligation: bead-bound sample was incubated with 45 μl buffer enzyme mix and 5 μl of different TruSeq DNA adapters (Illumina) for each sample, for 15 min at 20 °C, followed by PEG/NaCl purification (twice). Library enrichment: 12 cycles of PCR amplification. Size selection was performed after PCR using a 0.6×/0.8x ratio of Ampure XP beads (double size selection) set to collect 250–450 bp fragments. ChIP libraries were sequenced using Illumina HiSeq 2000 at 50 bp SE reads.

### ChIP and RNA-sequencing analysis

Single-end 50 bp ChIP-seq datasets were aligned using bwa-mem (version 0.7.15, default parameters) to 1 K genome hg19. Reads with identical start and end coordinates were discarded as PCR duplicates. Reads were then filtered for mapping quality of >5 and extended by 250 bp. Raw TSS-specific H3K27me3 RPKM calculations were then performed using SeqMonk using ENSEMBL gene annotation. TSS value was calculated in 3 kb centered bins on the transcription start site. H3K27me3 and H3K27ac RPKM values were then manually normalized to spike-in Rx values. The two technical replicates were then averaged. Non-specific enrichment in the input libraries were subtracted from the normalized ChIP RPKM values. *Z*-score was calculated from the mean RPKM as *z* = (Mutant–WT)/SQRT(|Mutant|+|WT|).

Total RNA was extracted from cell pellets and mouse tumors using the RNeasy mini kit (Qiagen) according to instructions from the manufacturer. Library preparation was performed with ribosomal RNA (rRNA) depletion according to instructions from the manufacturer (Epicentre) to achieve greater coverage of mRNA and other long non-coding transcripts. Paired-end sequencing was performed on the Illumina HiSeq 2500 platform. RNA-sequencing reads were stripped of adapter sequences using Trimmomatic (v0.32)^53^. Low-quality bases in the first four positions as well as at the end of each read were removed using a 4-bp sliding window average trim (average phred33 <30). An additional 3 bp was clipped from the start and end of a read if still found of low quality. Short reads (<30 bp) produced as a result of the trimming process were subsequently discarded. The remaining reads were aligned to the hg19 (GRCh37) build of the human genome using STAR (v2.3.0e)^54^ with default parameters. Reads with identical start and end coordinates were discarded as PCR duplicates. Reads were then filtered for mapping quality of >5. Gene expression RPKM were calculated using only strand-specific reads using SeqMonk using the ENSEMBL gene annotation. Triplicate RNA-seq were averaged prior to calculation of *z*-score. *Z*-score was calculated from the mean RPKM as *z* = (Mutant–WT)/SQRT(Mutant+WT). *P*-value was generated from a two-tailed *t*-test. Significant deregulation threshold for RNA-seq: |*z*| >1.5, *p*-value <0.05. Expanded analysis using a significant deregulation threshold of |*z*| >0.8, *p*-value <0.05. Significantly enriched Gene Ontology were called using PANTHER.

### Drug treatment

TEX cells were treated with either DMSO, UNC1999, GSK-J4, JQ1, panobinostat, vorinostat and trichostatin A at indicated concentrations. Drugs were obtained from Tocris and through the Structural Genomic Consortium. Cells were harvested and live cells (SYTOX®-negative) were counted using a BD LSRFortessa with an HTS (BD Biosciences).

### Statistical analysis

Unless otherwise stated, mean ± standard deviation values are given and p-values were calculated by two-tailed unpaired Student’s *t*-test. In the case of secondary transplantation, a Mann–Whitney test was used to compare engraftment between mice injected with wildtype or mutant cells. Boxplots were drawn using R. The center line is the median, the whiskers are minimum and maximum after removing outliers, the box limits are quartiles, and notches are ±1.58 IQR/sqrt(n) and generally represent a 95% confidence interval of median.

### Reporting summary

Further information on research design is available in the Nature Research Reporting Summary linked to this article.

## Supplementary information

Supplementary Information

Description of Additional Supplementary Files

Supplementary Data 1

Supplementary Data 2

Supplementary Data 3

Reporting Summary

Source Data

## Data Availability

Raw exome and transcriptome sequence data have been deposited at the European Genome-phenome Archive (EGA), which is hosted by the European Bioinformatics Institute (EMBLEBI) and the Centre for Genomic Regulation (CRG), under accession number EGAS00001003288 All associated ChIP- and RNA-seq data have been deposited to GEO under the GEO accession GSE122273.
