# Supplementary material for: Mutant H3 histones drive human pre-leukemic hematopoietic stem cell expansion and promote leukemic aggressiveness
Source: Nat Commun. 2019 Jun 28;10:2891. doi: 10.1038/s41467-019-10705-z (PMC6599207; doi:10.1038/s41467-019-10705-z)
Supplement: Supplementary file 1 — Supplementary Information [file 41467_2019_10705_MOESM1_ESM.pdf]

Supplementary Information

Mutated H3 Histones Drive Human Pre-Leukemic Hematopoietic Stem Cell Expansion

And Promote Leukemic Aggressiveness.

Boileau et al.

Karyotype	AUBMC<br>N (%)	Toronto<br>N (%)
Successful	108 (89)	292 (94)
No data	14 (11)	20 (6)
Normal	34 (31)	148 (51)
t (15;17)	7 (6)	11 (4)
t (8;21)	8 (7)	18 (6)
Inv (16)	7 (6)	15 (5)
t (9;11)	4 (4)	4 (1)
t (9;22)	2 (2)	1 (0)
t (6;9)	4 (4)	2 (1)
Hyper diploid	1 (1)	0 (0)
Complex	20 (19)	31 (11)
Other	40 (37)	62 (21)

Supplementary Table 1 Summary of cytogenetic information of the two cohorts sequenced for H3 histone mutations as determined by karyotypic analysis.

Molecular Abnormalities	AUBMC N (%)	Toronto N (%)
PML-RARA	11 (9)	NA
AML1-ET0	11 (9)	NA
CBF-MYH11	8 (7)	NA
AF9-MLL	4 (3)	NA
BCR-ABL	2 (2)	NA
NPM1c	14 (23)	67 (56)
FLT3-ITD	29 (24)	40 (30)
FLT3-D835	9 (7)	7 (6)
CEBPA	6 (14)	NA
CKIT	1 (2)	NA
IDH1	13 (11)	11 (17)
IDH2	8 (7)	13 (51)

Supplementary Table 2 Summary of abnormalities of the two cohorts sequenced for H3 histone mutations as determined by molecular assays.

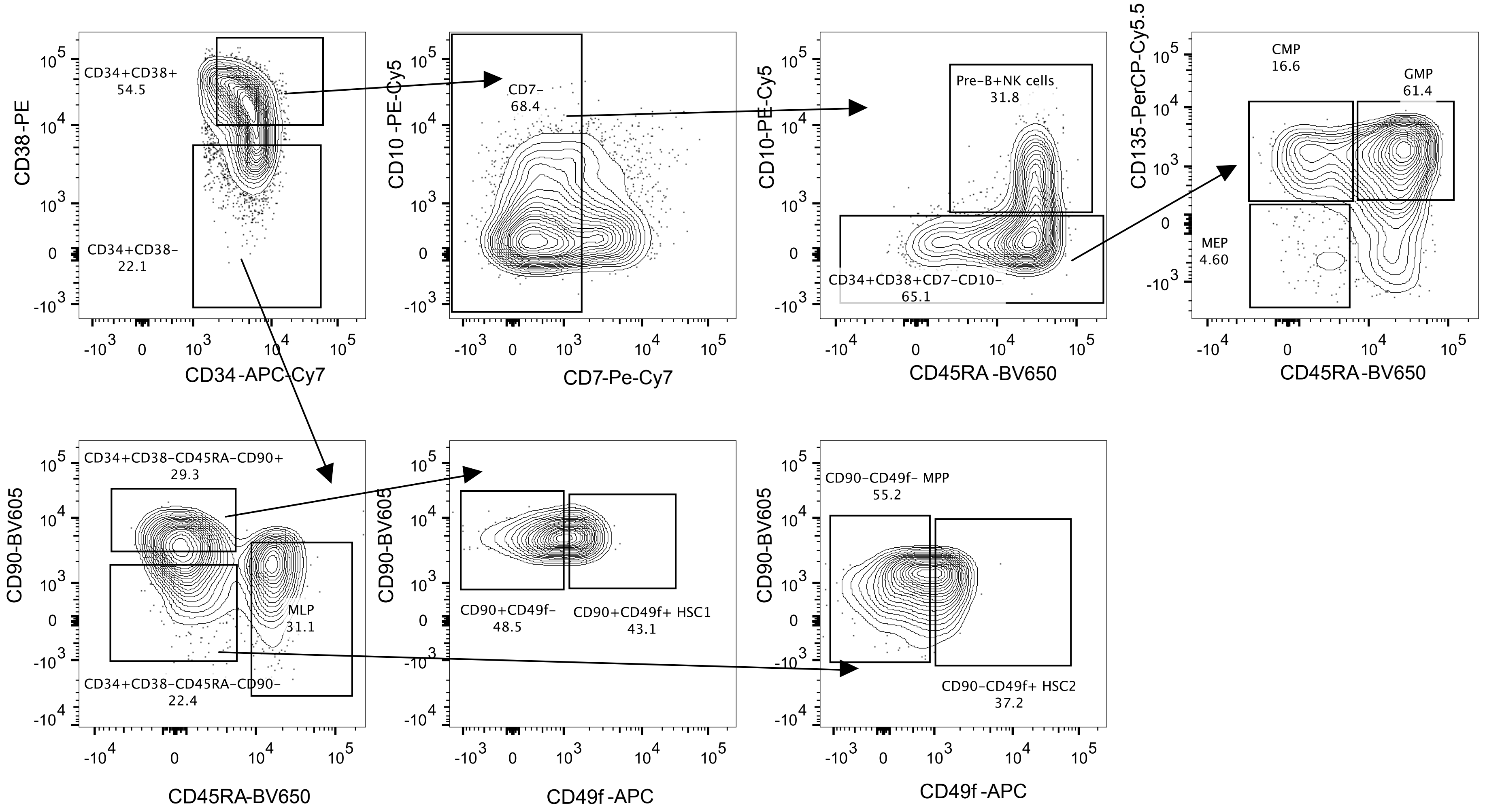

Supplementary Figure 1

Supplementary Figure 1 The gating scheme for defining HSC1(CD34+CD38-CD45RA-CD90+CD49f+), HSC2 (CD34+CD38-CD45RA-CD90-CD49f+), multipotent progenitor (MPP-CD34+CD38-CD90-CD49f-), multilymphoid progenitor (MLP-CD34+CD34-CD90-CD45RA+), common myeloid progenitor (CMP-CD34+CD38+CD7-CD10-CD135+CD45RA-), megakaryocyte-erythroid progenitor (MEP-CD34+CD38+CD7-CD10-CD135-CD45RA-) and granulocyte-macrophage progenitor (GMP-CD34+CD38+CD7-CD10-CD135+CD45RA+) from a representative lineage and mouse depleted sample of the bone marrow of a mouse xenotransplanted with cord blood cells overexpressing HIST1H3H K27M.

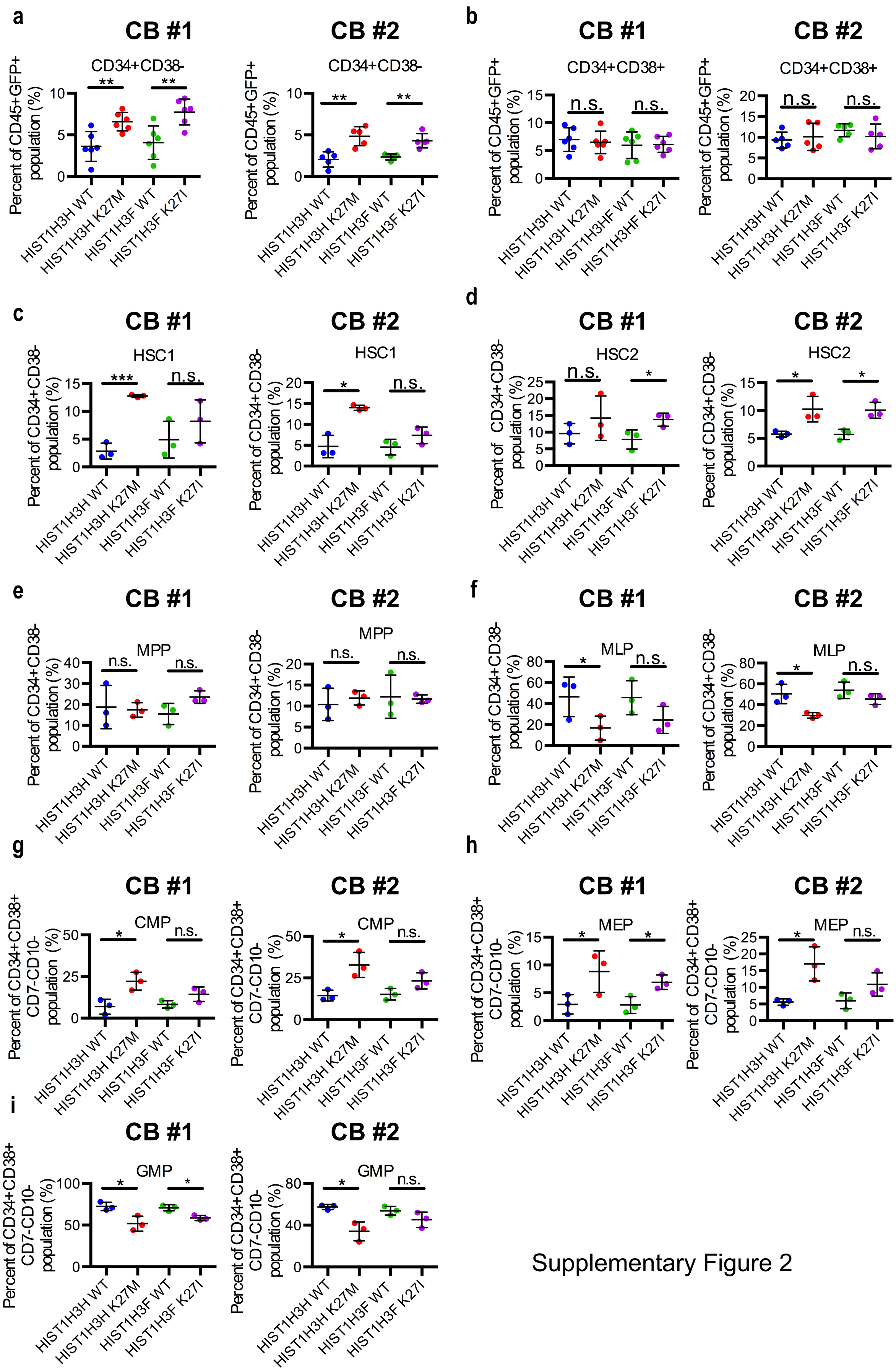

Supplementary Figure 2

Supplementary Figure 2 H3.1 K27 mutations alter HSC frequency and hematopoietic differentiation *in vivo*. Flow cytometry analysis of **(a)** CD34+CD38- cells, **(b)** CD34+CD38+ cells, **(c)** HSC1, **(d)** HSC2, **(e)** MPP, **(f)** MLP, **(g)** CMP, **(h)** MEP and **(l)** GMP populations from the bone marrow of the injected femur of mice injected with CD34+CD38- human cord blood transduced with HIST1H3H WT/K27M or HIST1H3F WT/K27I after 12-14 weeks. Both biologically replicated experiments are shown (CB#1 and CB#2). Data represents the mean and the error bars are the standard deviation.; n=6 (CB#1), n=5 (CB#2). Statistical analysis was performed by two-way Student's t-tests. *$p \leq 0.05$, **$p \leq 0.01$, ***$p \leq 0.005$, ****$p \leq 000.1$

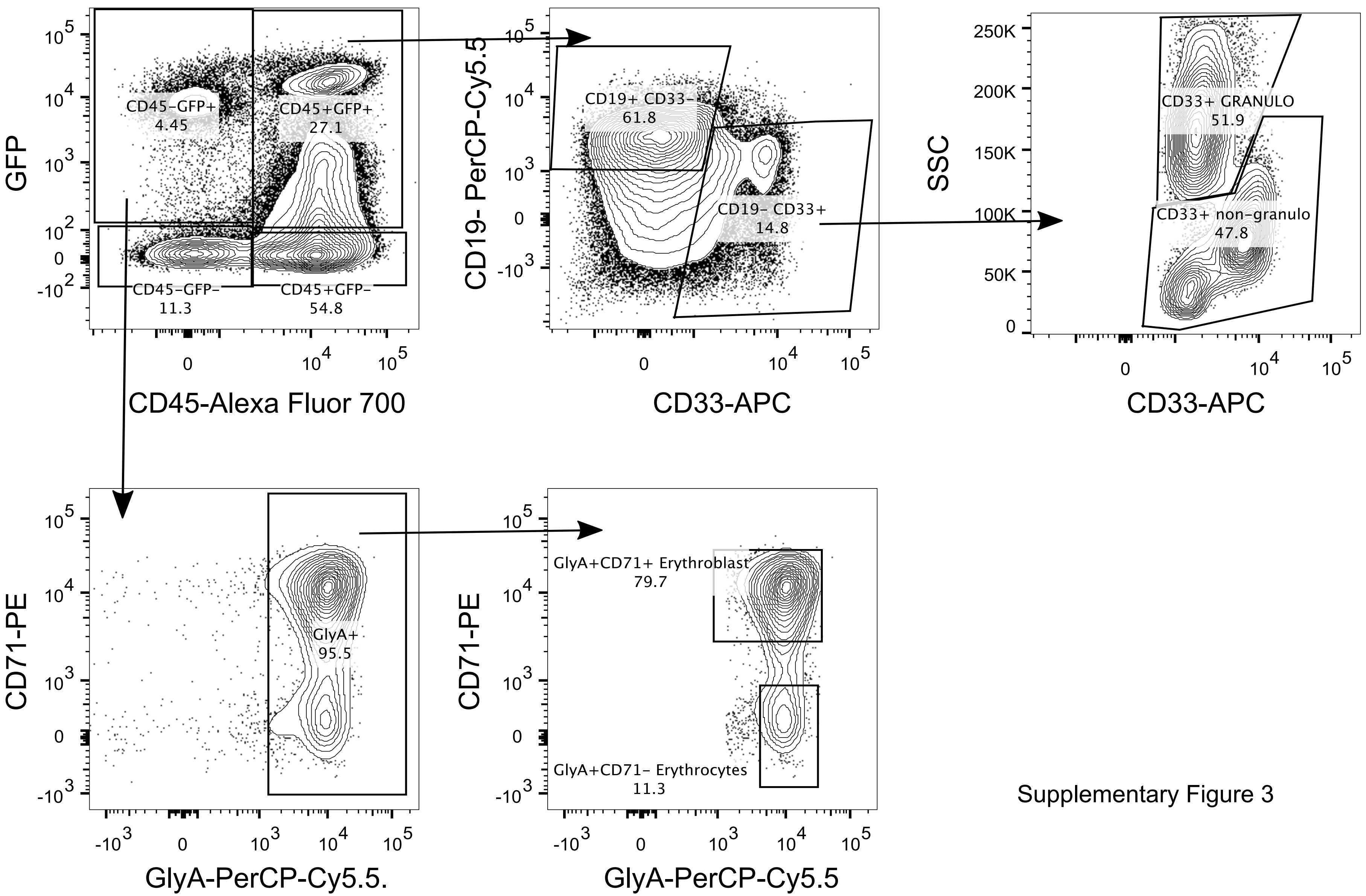

Supplementary Figure 3

Supplementary Figure 3 The gating scheme for defining granulocytes (CD45+CD33+SSChigh), erythroblasts (CD45-GlyA+CD71+) and erythrocytes (CD45-GlyA+CD71-) from a representative sample of the bone marrow of a mouse xenotransplanted with CD34+CD38- cord blood cells overexpressing HIST1H3H K27M.

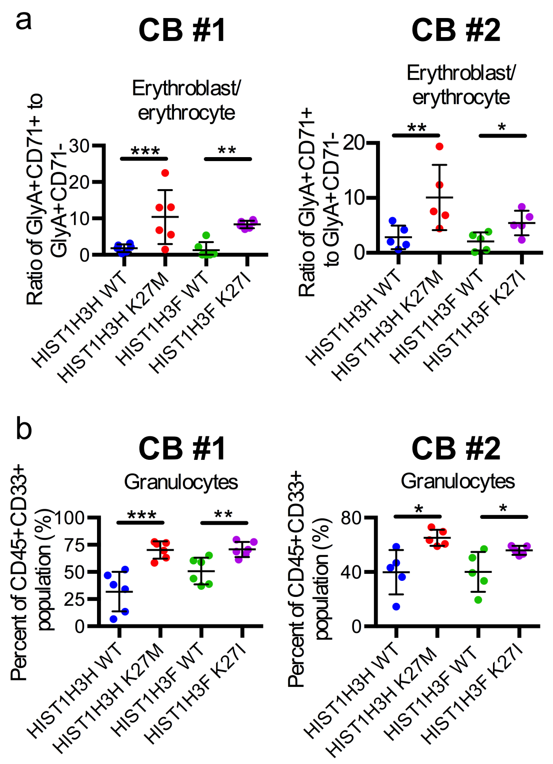

Supplementary Figure 4 H3.1 K27M/I mutations alter erythroid and granulocyte differentiation of human cord blood. Flow cytometry analysis of **(a)** erythroid and **(b)** granulocyte populations from the bone marrow of the injected femur of mice xenotransplanted with CD34+CD38- human cord blood cells transduced with HIST1H3H WT/K27M or HIST1H3F WT/K27I after 12-14 weeks. Both biologically replicated experiments are shown (CB#1 and CB#2). Data represents the mean and error bars are the standard deviations; n=6 (CB#1), n=5(CB #2). Statistical analysis was performed by two-way Student's t-tests. *p≤0.05, **p≤0.01, ***p≤0.001, ***p≤0.0001.

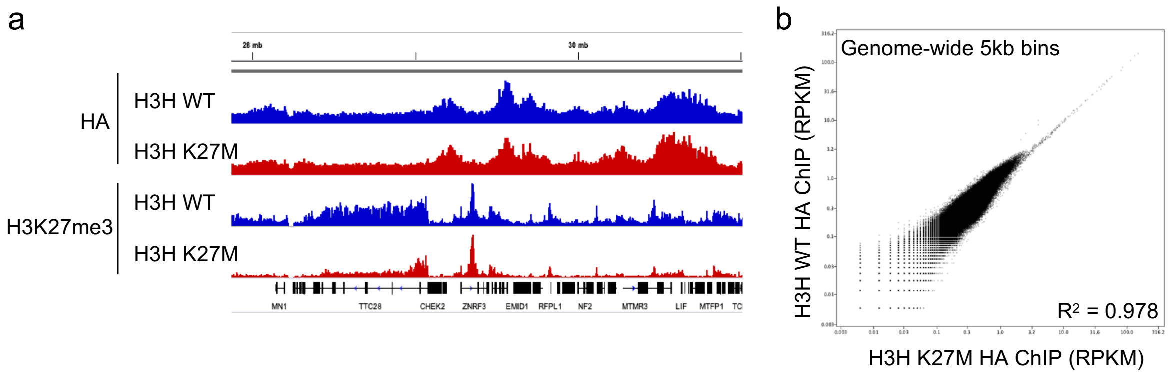

Supplementary Figure 5 DNA localization of transduced HIST1H3H K27M and wild type histone. **(a)** Genome browser screenshot showing similar enrichment pattern between WT and K27M histone using HA-tagged ChIP. **(b)** Pair-wise correlation of HA-tagged ChIP RPKM in WT vs. K27M TEX cells. R^2 = Pearson's correlation.

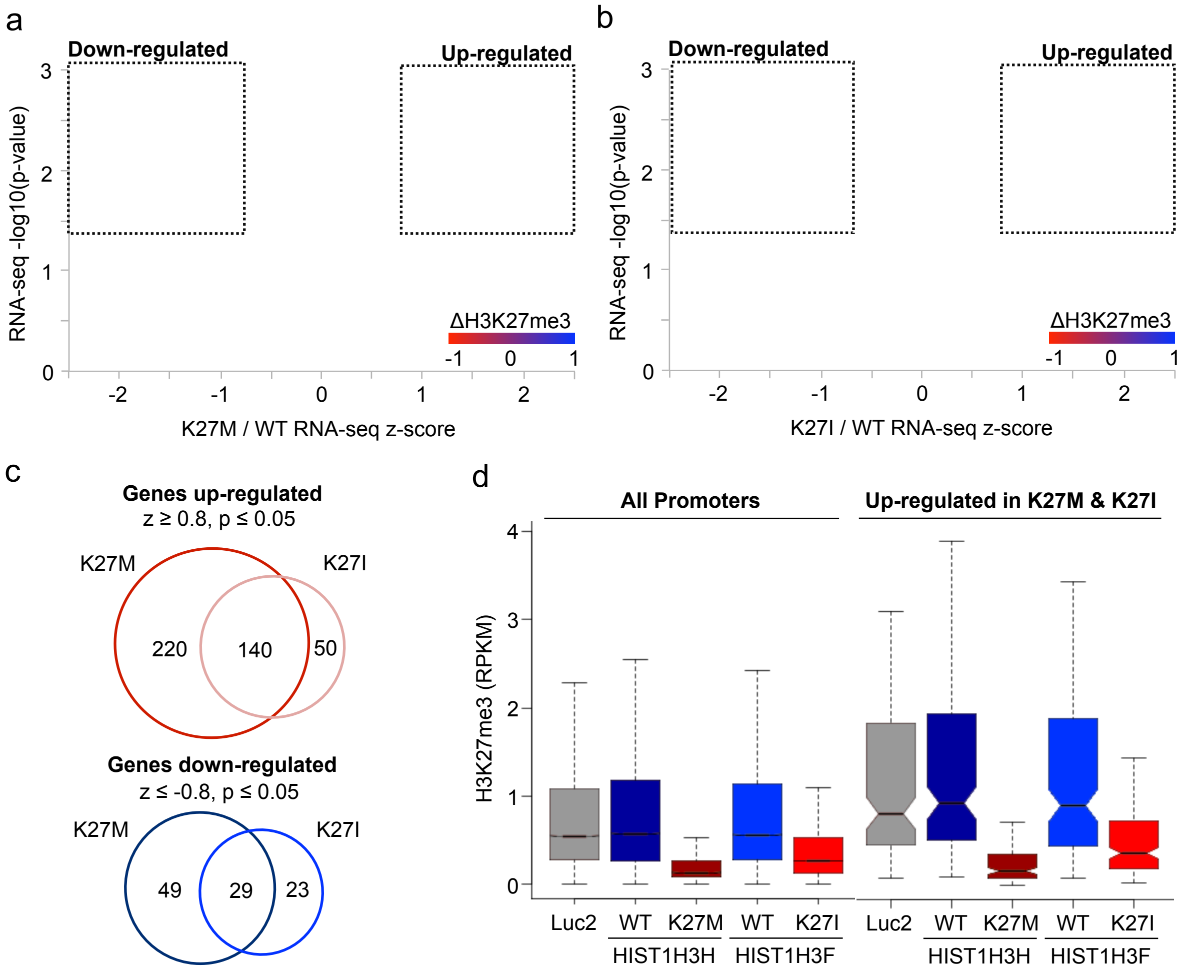

e

GO biological process (genes up-regulated in K27M)	Hits	FDR p-value
negative regulation of cell activation (GO:0050866)	14/185	4.57E-02
positive regulation of cytosolic calcium ion concentration (GO:0007204)	17/268	4.30E-02
neutrophil degranulation (GO:0043312)	29/483	6.06E-05
regulation of leukocyte activation (GO:0002694)	31/572	1.72E-04
regulation of inflammatory response (GO:0050727)	22/410	2.44E-02
chemotaxis (GO:0006935)	25/519	3.20E-02
cell adhesion (GO:0007155)	38/906	3.68E-03
regulation of nervous system development (GO:0051960)	25/883	2.84E-02
regulation of multicellular organismal development (GO:2000026)	69/1918	1.69E-05

GO biological process (genes down-regulated in K27M)	Hits	FDR p-value
interferon-gamma-mediated signaling pathway (GO:0060333)	8/71	1.81E-06
antigen processing and presentation of exogenous peptide antigen via MHC class II (GO:0019886)	6/98	1.08E-02
neutrophil degranulation (GO:0043312)	10/483	4.30E-02
response to bacterium (GO:0009617)	12/644	1.30E-02
cell adhesion (GO:0007155)	14/906	1.38E-02

Supplementary Figure 6

Supplementary Figure 6. Expanded analysis of gene expression changes using a z-score cut-off of ±0.8. **(a)**Volcano plot depicting genes showing differential expression in K27M relative to HIST1H3H WT and **(b)**K27I relative to HIST1H3F WT. H3K27me3 change of K27M/WT and K27I/WT is overlaid as a heatmap, with red and blue representing loss and gain, respectively. Dashed gates indicate genes called as being significantly down- or up-regulated, using threshold of |z-score|≥0.8, p-value≤0.05. **(c)**Venn diagram showing overlap of significantly up- and down-regulated genes observed in K27M and K27I cells. **(d)**Box-whisker plot showing change of promoter-specific H3K27me3 in the TEX cells, comparing all annotated genes. The lower and upper whisker represents the minimum and maximum, respectively, after removing outliers where the upper whisker = min(max(x), Q3 + 1.5 * IQR) and lower whisker = max(min(x), Q1 - 1.5 * IQR), where IQR = Q3-Q1. The two 'hinges' are versions of the first and third quartile. The notches extend to +/-1.58 IQR/sqrt(n) representing a confidence interval. Center line indicates median. Note that the up-regulated genes show significantly higher level of H3K27me3 at the promoter in the WT compared to the average promoter. **(e)** Gene ontology enrichment analysis of significantly up- and down-regulated-genes in K27M / WT. Hits represent the total number of genes identified over the total number of genes annotated for the specific GO term. FDR: Fisher's exact test, corrected for multiple testing.

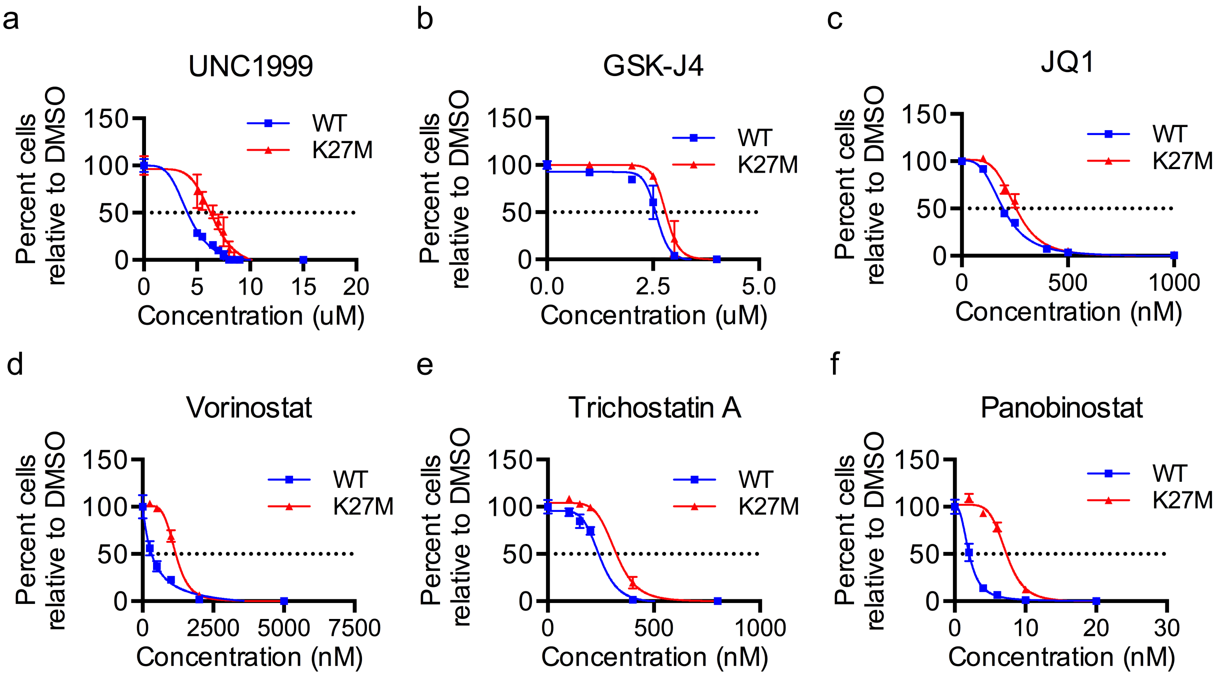

Supplementary Figure 7 TEX cells overexpressing H3.1 K27M are resistant to compounds that modulate histone methylation and acetylation. **(a-f)** TEX cells overexpressing HIST1H3H WT or K27M were treated with **(a)** UNC1999, **(b)** GSK-J4, **(c)** JQ1, **(d)** vorinostat, **(e)** trichostatin A, and **(f)** panobinostat for 5 days at the indicated concentrations. Viability was assessed by flow cytometry. Data represents the mean and error bars are standard deviation; n=3 and is representative of at least 2 independent experiments.
