# Supplementary material for: Mutant H3 histones drive human pre-leukemic hematopoietic stem cell expansion and promote leukemic aggressiveness
Source: Nat Commun. 2019 Jun 28;10:2891. doi: 10.1038/s41467-019-10705-z (PMC6599207; doi:10.1038/s41467-019-10705-z)
Supplement: Supplementary file 2 — Description of Additional Supplementary Files [file 41467_2019_10705_MOESM2_ESM.pdf]

Description of Additional Supplementary Information

File Name: Supplementary Data 1
Description: Complete RNA-seq, H3K27me3 ChIP-seq and H3K27ac ChIP-seq data for TEX cells transduced with Luc2, HIST1H3H WT, HIST1H3H K27M, HIST1H3F WT or HIST1H3F K27I and untransduced cells. Fold-change (FC), z-scores and p-values for gene expression, H3K27me3 and H3K27ac are shown. This data was used to generate plots shown in Figure 3b-g and Supplementary Figure 6a-d.

File Name: Supplementary Data 2
Description: Significantly altered genes between HIST1H3H WT and K27M and HIST1H3F WT and K27I.

File Name: Supplementary Data 3
Description: ChIP-seq data HA-tag for localization of HA-tagged H3.1 histones in TEX cells transduced with HIST1H3H WT or HIST1H3H. This data was used to generate plots in Supplementary Figure 5.
