# Supplementary material for: Mutant H3 histones drive human pre-leukemic hematopoietic stem cell expansion and promote leukemic aggressiveness
Source: Nat Commun. 2019 Jun 28;10:2891. doi: 10.1038/s41467-019-10705-z (PMC6599207; doi:10.1038/s41467-019-10705-z)
Supplement: Supplementary file 6 — Reporting Summary [file 41467_2019_10705_MOESM6_ESM.pdf]

nature research

Corresponding author(s): Dr. Kolja Eppert

Reporting Summary

Nature Research wishes to improve the reproducibility of the work that we publish. This form provides structure for consistency and transparency in reporting. For further information on Nature Research policies, see Authors & Referees and the Editorial Policy Checklist.

Statistical parameters

When statistical analyses are reported, confirm that the following items are present in the relevant location (e.g. figure legend, table legend, main text, or Methods section).

n/a	Confirmed	
☐	☒	The exact sample size (n) for each experimental group/condition, given as a discrete number and unit of measurement
☐	☒	An indication of whether measurements were taken from distinct samples or whether the same sample was measured repeatedly
☐	☒	The statistical test(s) used AND whether they are one- or two-sided<br>*Only common tests should be described solely by name; describe more complex techniques in the Methods section.*
☒	☐	A description of all covariates tested
☐	☒	A description of any assumptions or corrections, such as tests of normality and adjustment for multiple comparisons
☐	☒	A full description of the statistics including central tendency (e.g. means) or other basic estimates (e.g. regression coefficient) AND variation (e.g. standard deviation) or associated estimates of uncertainty (e.g. confidence intervals)
☐	☒	For null hypothesis testing, the test statistic (e.g. F, t, r) with confidence intervals, effect sizes, degrees of freedom and P value noted<br>*Give P values as exact values whenever suitable.*
☒	☐	For Bayesian analysis, information on the choice of priors and Markov chain Monte Carlo settings
☒	☐	For hierarchical and complex designs, identification of the appropriate level for tests and full reporting of outcomes
☒	☐	Estimates of effect sizes (e.g. Cohen's d, Pearson's r), indicating how they were calculated
☐	☒	Clearly defined error bars<br>*State explicitly what error bars represent (e.g. SD, SE, CI)*

Our web collection on statistics for biologists may be useful.

Software and code

Policy information about availability of computer code

Data collection	DIVA version 8.0 (build 2013 07 02 02 11)

Data analysis	Prism version 6.00 for Mac, GraphPad Software (generation of graphs)<br>FlowJo version x 10.0.7r2 and 10.1r5 (analysis of FCS files)<br>Excel for mac 2011<br>Burrows-Wheeler Aligner (aligning whole exome sequencing reads)<br>Picard http://broadinstitute.github.io/picard/ (remove duplicate reads from whole exome sequencing reads)<br>SAMtools Mpileup (variant calling)<br>FreeBayes v1.1.0-4-gb6041c6 (variant calling)<br>GATK haplotype caller version 3.8 (variant calling)<br>ANNOVAR (annotating mutations)<br>BWA-mem version 0.7.15 (aligning ChIP-seq reads)<br>SeqMonk (ChIP-seq and RNA-seq RPKM calculations)<br>Trimmomatic v0.32 (stripping adaptor sequences from RNA-seq reads)<br>STAR v2.3.0e (aligning RNA-seq reads)<br>PANTHER (Gene Ontology Analysis)

For manuscripts utilizing custom algorithms or software that are central to the research but not yet described in published literature, software must be made available to editors/reviewers upon request. We strongly encourage code deposition in a community repository (e.g. GitHub). See the Nature Research guidelines for submitting code & software for further information.

Data

Policy information about availability of data

All manuscripts must include a data availability statement. This statement should provide the following information, where applicable:

- Accession codes, unique identifiers, or web links for publicly available datasets
- A list of figures that have associated raw data
- A description of any restrictions on data availability

"Raw exome and transcriptome sequence data have been deposited at the European Genome-phenome Archive (EGA), which is hosted by the European Bioinformatics Institute (EMBLEBI) and the Centre for Genomic Regulation (CRG), under accession number EGAS00001003288". All associated ChIP- and RNA-seq (Fig. 3) have been deposited to GEO, set to be release on Nov. 6, 2019 or upon acceptance of publication, whichever comes first.

Field-specific reporting

Please select the best fit for your research. If you are not sure, read the appropriate sections before making your selection.

☒ Life sciences ☐ Behavioural & social sciences ☐ Ecological, evolutionary & environmental sciences

For a reference copy of the document with all sections, see nature.com/authors/policies/ReportingSummary-flat.pdf

Life sciences study design

All studies must disclose on these points even when the disclosure is negative.

Sample size	Sample size was not calculated for the exploratory analysis of histone mutations in AML as the full set of samples available in two cohorts was examined. For in vivo experiments, the number of mice per condition was estimated based on standard approaches (e.g. 3-6 mice per condition is sufficient to detect significant alterations in hematopoiesis or leukemic growth).
Data exclusions	One mouse was excluded from Figure 2d-g from the HIST1H3H K27M group due a missed intrafemoral injection as confirmed by an excessively large mass around the injection site and lack of substantial engraftment.
Replication	Replicates are described in the text and each reproduced the data. In summary, in each case 3 or more replicates were included per experiment and experiments were repeated as completely independent experiments two or more times, including starting from new viral transductions of cells.
Randomization	Mice were allocated to the different experimental conditions randomly. Mice in the same cage (same litter) were randomly distributed to new cages one week prior to performing the experiments.
Blinding	The investigators were blinded to AML sample/patient characteristics during sequence analysis. They were not blinded during in vivo mouse xenotransplantation experiments.

Reporting for specific materials, systems and methods

Materials & experimental systems

n/a	Involved in the study
☐	☒ Unique biological materials
☐	☒ Antibodies
☐	☒ Eukaryotic cell lines
☒	☐ Palaeontology
☐	☒ Animals and other organisms
☐	☒ Human research participants

Methods

n/a	Involved in the study
☐	☒ ChIP-seq
☐	☒ Flow cytometry
☒	☐ MRI-based neuroimaging

Unique biological materials

Policy information about availability of materials

Obtaining unique materials

Availability off biological materials is restricted. Please contact corresponding author (kolja.eppert@mcgill.ca) if you wish to obtain any specific material used in the manuscript.

Antibodies

Antibodies used

Standard antibodies were used for each cell population.

Antibody Fluorochrome Clone Supplier Catalog # Size Date Lot:

CD3 BV605 SK7 Biolegend 344836 100 tests 31-03-2017 Lot: B229167

CD7 PE-Cy7 CD7-6B7 Biolegend 343113 25 tests 29-08-2016 Lot: B184809

CD10 PE-Cy5 HI10a Biolegend 312206 100 tests 20-12-2016 Lot: B200344

CD14 PE-Dazzle HCD14 Biolegend 325634 100 tests 31-03-2017 Lot: B207835

CD19 PerCP-Cy5.5 HIB19 Biolegend 302230 100 tests 27-04-2015 Lot: B183463
02-12-2015 Lot: B183463
08-06-2016 Lot: B183463

CD33 APC WM53 Biolegend 303408 100 tests 27-04-2015 Lot: B195978
02-12-2015 Lot: B195978
08-06-2016 Lot: B203959

CD34 APC 581 Biolegend 343510 100 tests 21-09-2015 Lot: B186978
08-06-2016 Lot: B186978
06-04-2017 Lot: B234734

CD34 APC-Cy7 581 Biolegend 343514 100 tests 27-04-2015 Lot: B195545
02-12-2015 Lot: B195545

CD38 PE HB-7 Biolegend 356604 100 tests 13-02-2015 Lot: B178346
21-09-2015 Lot: B178346
08-06-2016 Lot: B178346
31-08-2016 Lot: B178346

CD41 PE-Cy7 HIP8 Biolegend 303718 100 tests 31-03-2017 Lot: B225898

CD45 Alexa Fluor 700 2D1 Biolegend 368514 100 tests 20-12-2016 Lot: B218661
31-03-2017 Lot: B230419
27-05-2017 Lot: B230419

CD45RA BV650 HI100 Biolegend 304135 100 tests 29-08-2016 Lot: B216958
16-06-2017 Lot: B234406

CD49f APC GoH3 Biolegend 313616 100 tests 20-12-2016 Lot: B211208

CD56 (NCAM) PE-Cy5 hCD56 Biolegend 318308 100 tests 31-03-2017 Lot: B222359

CD71 PE M-A712 BD Biosciences 555537 100 tests 13-04-2017 Lot: 6210902

CD90 (Thy1) BV605 5E10 Biolegend 328128 100 tests 29-08-2016 Lot: B209021

CD135 (Flt-3/Flk-2) PerCP-Cy5.5 BV10A4H2 Biolegend 313316 100 tests 29-08-2016 Lot: B200971
16-06-2017 Lot: B221395

CD235a (GlyA) PerCP-Cy5.5 HI264 Biolegend 349110 100 tests 27-04-2015 Lot: B189995
20-12-2016 Lot: B220906
27-05-2017 Lot: B220906

For ChIP, the following antibodies were used: anti-H3K27me3 (Active Motif, 61017), anti-H3K27ac (Diagenode C15410196) and anti-HA-tag (Cell Signaling Technologies, 3724).

Validation

Please refer to www.biolegend.com and www.bdbiosciences.com for the "Technical Data Sheet" describing the product details.

For ChIP, the specificity of antibodies was tested by dot blot on a histone modification peptide array. Antibodies used have been used in the literature and also validated by manufacturers.

Eukaryotic cell lines

Policy information about cell lines

Cell line source(s)

TEX cells: available from the laboratory of Dr. John E. Dick.

Authentication

Not authenticated through finger printing. Flow cytometry was used to confirm immunophenotype matched the published work first describing TEX cells (Warner et al. Leukemia, 2005).

Mycoplasma contamination

Cells were routinely tested for mycoplasma using the MycoAlert mycoplasma testing kit from Lonza (catalog # LT07-218)

Commonly misidentified lines
(See ICLAC register)

N/A

Animals and other organisms

Policy information about studies involving animals; ARRIVE guidelines recommended for reporting animal research

Laboratory animals

8-16 week old female NOD-scid IL2Rgnull (NSG) and NSG-SGM3 mice were used as indicated. The animal use protocol was approved by McGill University.

Wild animals

N/A

Field-collected samples

N/A

Human research participants

Policy information about studies involving human research participants

Population characteristics

DNA from leukemic samples from human AML patients was used in this study. Two cohorts were included in this study. 122 DNA samples were from the American University of Beirut Medical Centre where the mean age of AML patients was 32.2 (0-83). 312 samples were collected at the University Heath Network in Toronto Canada where the mean age of AML patients was 52.7 (18-82).

Recruitment

Bone marrow samples at AUBMC were obtained from AML patients who had their DNA previously collected for routine diagnostic purposes and stored in the Department of Pathology and Laboratory Medicine as part of the College of American pathologist accreditation requirements from January 2005 to January 2015. All patients from the Toronto Cohort were recruited with informed consent according to procedures approved by the Research Ethics Board of the University Health Network (UHN; REB# 01-0573-C) and samples viably frozen in the PM Leukaemia Bank.

ChIP-seq

Data deposition

☒ Confirm that both raw and final processed data have been deposited in a public database such as GEO.

☐ Confirm that you have deposited or provided access to graph files (e.g. BED files) for the called peaks.

Data access links
May remain private before publication.

https://www.ncbi.nlm.nih.gov/geo/query/acc.cgi?acc=GSE122273

Files in database submission

HI.4591.7.Index_8.TEX-HIST1H3F-K27I-2-Rx_cells_ChIP1_H3K27ac_1.bam
HI.4591.7.Index_23.TEX-HIST1H3F-K27I-Rx_cells_ChIP1_H3K27ac_1.bam
HI.3774.6.Index_4.TEX-HIST1H3F-K27I-Rx_cells_ChIP1_H3K27me3_1.bam
HI.3775.2.Index_12.TEX-HIST1H3F-K27I-Rx_cells_ChIP1_Input_1.bam
HI.4591.7.Index_3.TEX-HIST1H3F-WT-2-Rx_cells_ChIP1_H3K27ac_1.bam
HI.4591.7.Index_22.TEX-HIST1H3F-WT-Rx_cells_ChIP1_H3K27ac_1.bam
HI.3774.6.Index_2.TEX-HIST1H3F-WT-Rx_cells_ChIP1_H3K27me3_1.bam
HI.3775.2.Index_7.TEX-HIST1H3F-WT-Rx_cells_ChIP1_Input_1.bam

HI.4591.7.Index_20.TEX-HIST1H3H-K27M-2-Rx_cells_ChIP1_H3K27ac_1.bam
HI.4591.7.Index_27.TEX-HIST1H3H-K27M-Rx_cells_ChIP1_H3K27ac_1.bam
HI.3774.7.Index_6.TEX-HIST1H3H-K27M-Rx_cells_ChIP1_H3K27me3_1.bam
HI.3774.7.Index_14.TEX-HIST1H3H-K27M-Rx_cells_ChIP1_Input_1.bam
HI.4591.7.Index_10.TEX-HIST1H3H-WT-2-Rx_cells_ChIP1_H3K27ac_1.bam
HI.4591.7.Index_25.TEX-HIST1H3H-WT-Rx_cells_ChIP1_H3K27ac_1.bam
HI.3774.6.Index_5.TEX-HIST1H3H-WT-Rx_cells_ChIP1_H3K27me3_1.bam
HI.3775.2.Index_13.TEX-HIST1H3H-WT-Rx_cells_ChIP1_Input_1.bam
HI.4591.7.Index_1.TEX-Luciferase-2-Rx_cells_ChIP1_H3K27ac_1.bam
HI.4591.7.Index_21.TEX-Luciferase-Rx_cells_ChIP1_H3K27ac_1.bam
HI.3774.7.Index_7.TEX-Luciferase-Rx_cells_ChIP1_H3K27me3_1.bam
HI.3774.7.Index_15.TEX-Luciferase-Rx_cells_ChIP1_Input_1.bam
TEX_H3-1F_K27I.bam
TEX_H3-1F_K27I_1.bam
TEX_H3-1F_K27I_2.bam
TEX_H3-1F_WT.bam
TEX_H3-1F_WT_1.bam
TEX_H3-1F_WT_2.bam
TEX_H3-1H_K27M.bam
TEX_H3-1H_K27M_1.bam
TEX_H3-1H_K27M_2.bam
TEX_H3-1H_WT.bam
TEX_H3-1H_WT_1.bam
TEX_H3-1H_WT_2.bam
TEX_Luc2.bam
TEX_Luc2_1.bam
TEX_Luc2_2.bam
TEX_UT.bam
TEX_UT_1.bam
TEX_UT_2.bam
TEX-HIST1H3F-K27I-2-Rx_cells_ChIP1_H3K27ac_1.bw
TEX-HIST1H3F-K27I-Rx_cells_ChIP1_H3K27ac_1.bw
TEX-HIST1H3F-WT-Rx_cells_ChIP1_H3K27me3_1.bw
TEX-HIST1H3F-K27I-Rx_cells_ChIP1_Input_1.bw
TEX-HIST1H3F-WT-2-Rx_cells_ChIP1_H3K27ac_1.bw
TEX-HIST1H3F-WT-Rx_cells_ChIP1_H3K27ac_1.bw
TEX-HIST1H3F-WT-Rx_cells_ChIP1_H3K27me3_1.bw
TEX-HIST1H3F-WT-Rx_cells_ChIP1_Input_1.bw
TEX-HIST1H3H-K27M-2-Rx_cells_ChIP1_H3K27ac_1.bw
TEX-HIST1H3H-K27M-Rx_cells_ChIP1_H3K27ac_1.bw
TEX-HIST1H3H-K27M-Rx_cells_ChIP1_H3K27me3_1.bw
TEX-HIST1H3H-K27M-Rx_cells_ChIP1_Input_1.bw
TEX-HIST1H3H-WT-2-Rx_cells_ChIP1_H3K27ac_1.bw
TEX-HIST1H3H-WT-Rx_cells_ChIP1_H3K27ac_1.bw
TEX-HIST1H3H-WT-Rx_cells_ChIP1_H3K27me3_1.bw
TEX-HIST1H3H-WT-Rx_cells_ChIP1_Input_1.bw
TEX-Luciferase-2-Rx_cells_ChIP1_H3K27ac_1.bw
TEX-Luciferase-Rx_cells_ChIP1_H3K27ac_1.bw
TEX-Luciferase-Rx_cells_ChIP1_H3K27me3_1.bw
TEX-Luciferase-Rx_cells_ChIP1_Input_1.bw
TEX_H3-1F_K27I.sorted.bw
TEX_H3-1F_K27I_1.sorted.bw
TEX_H3-1F_K27I_2.sorted.bw
TEX_H3-1F_WT.sorted.bw
TEX_H3-1F_WT_1.sorted.bw
TEX_H3-1F_WT_2.sorted.bw
TEX_H3-1H_K27M.sorted.bw
TEX_H3-1H_K27M_1.sorted.bw
TEX_H3-1H_K27M_2.sorted.bw
TEX_H3-1H_WT.sorted.bw
TEX_H3-1H_WT_1.sorted.bw
TEX_H3-1H_WT_2.sorted.bw
TEX_Luc2.sorted.bw
TEX_Luc2_1.sorted.bw
TEX_Luc2_2.sorted.bw
TEX_UT.sorted.bw
TEX_UT_1.sorted.bw
TEX_UT_2.sorted.bw
AML.ENSEMBL

Genome browser session
(e.g. UCSC)

NA

Methodology

Replicates

Two biological replicates were used in the analysis and both replicates are within agreement. See supplemental table 3 for RPKM reads.

Sequencing depth

Single-end, 50bp.
HI.3774.006.Index_2.TEX-HIST1H3F-WT-Rx_cells_ChIP1_H3K27me3_1.bam 64395171
HI.3774.006.Index_4.TEX-HIST1H3F-K27I-Rx_cells_ChIP1_H3K27me3_1.bam 69406508
HI.3774.006.Index_5.TEX-HIST1H3H-WT-Rx_cells_ChIP1_H3K27me3_1.bam 55252336
HI.3774.007.Index_14.TEX-HIST1H3H-K27M-Rx_cells_ChIP1_Input_1.bam 38402292
HI.3774.007.Index_15.TEX-Luciferase-Rx_cells_ChIP1_Input_1.bam 44741401
HI.3774.007.Index_6.TEX-HIST1H3H-K27M-Rx_cells_ChIP1_H3K27me3_1.bam 60218286
HI.3774.007.Index_7.TEX-Luciferase-Rx_cells_ChIP1_H3K27me3_1.bam 45915985
HI.3775.002.Index_12.TEX-HIST1H3F-K27I-Rx_cells_ChIP1_Input_1.bam 36115520
HI.3775.002.Index_13.TEX-HIST1H3H-WT-Rx_cells_ChIP1_Input_1.bam 42077499
HI.3775.002.Index_7.TEX-HIST1H3F-WT-Rx_cells_ChIP1_Input_1.bam 47916285
HI.4591.007.Index_10.TEX-HIST1H3H-WT-2-Rx_cells_ChIP1_H3K27ac_1.bam 36600973
HI.4591.007.Index_1.TEX-Luciferase-2-Rx_cells_ChIP1_H3K27ac_1.bam 35139391
HI.4591.007.Index_20.TEX-HIST1H3H-K27M-2-Rx_cells_ChIP1_H3K27ac_1.bam 32143500
HI.4591.007.Index_21.TEX-Luciferase-Rx_cells_ChIP1_H3K27ac_1.bam 25657459
HI.4591.007.Index_22.TEX-HIST1H3F-WT-Rx_cells_ChIP1_H3K27ac_1.bam 36528097
HI.4591.007.Index_23.TEX-HIST1H3F-K27I-Rx_cells_ChIP1_H3K27ac_1.bam 38171781
HI.4591.007.Index_25.TEX-HIST1H3H-WT-Rx_cells_ChIP1_H3K27ac_1.bam 38855898
HI.4591.007.Index_27.TEX-HIST1H3H-K27M-Rx_cells_ChIP1_H3K27ac_1.bam 34229434
HI.4591.007.Index_3.TEX-HIST1H3F-WT-2-Rx_cells_ChIP1_H3K27ac_1.bam 33180875
HI.4591.007.Index_8.TEX-HIST1H3F-K27I-2-Rx_cells_ChIP1_H3K27ac_1.bam 30536417

Paired-end, 100bp.
TEX_H3-1F_K27I_1.bam 138242524
TEX_H3-1F_K27I_2.bam 147177202
TEX_H3-1F_K27I.bam 149441772
TEX_H3-1F_WT_1.bam 152388938
TEX_H3-1F_WT_2.bam 166727506
TEX_H3-1F_WT.bam 139427066
TEX_H3-1H_K27M_1.bam 135001030
TEX_H3-1H_K27M_2.bam 165414320
TEX_H3-1H_K27M.bam 146983602
TEX_H3-1H_WT_1.bam 145017964
TEX_H3-1H_WT_2.bam 132613502
TEX_H3-1H_WT.bam 158603836
TEX_Luc2_1.bam 144152350
TEX_Luc2_2.bam 115565910
TEX_Luc2.bam 154715554
TEX_UT_1.bam 181396246
TEX_UT_2.bam 154804376
TEX_UT.bam 139769952

Antibodies

For ChIP, the following antibodies were used: anti-H3K27me3 (Active Motif, 61017), anti-H3K27ac (Diagenode C15410196) and anti-HA-tag (Cell Signaling Technologies, 3724).

Peak calling parameters

NA

Data quality

ChIP-sequencing experiments were assessed for their percent of mapped reads (to hg19 and dm6, when applicable) to ensure proper coverage. Reads with poor mapping quality were discarded from further analysis. Antibody pulldown efficacy was visually assessed by looking at tracks and comparing with spiked-in distribution of marks, when applicable.

Software

Single-end 50bp ChIP-seq datasets were aligned using bwa-mem (version 0.7.15, default parameters), to hg19. Reads with identical start and end coordinates were discarded as PCR duplicates. Reads were then filtered for mapping quality of > 5, and extended by 250bp. Raw TSS-specific H3K27me3 RPKM calculation were then performed using SeqMonk (version 1.42.0) using ENSEMBL gene annotation. TSS value is calculated in 3kb centred bins on the transcription start site. H3K27me3 and H3K27ac RPKM values were then manually normalized to spike-in Rx values. The two technical replicates were then averaged. Non-specific enrichment in the input libraries were subtracted from the normalized ChIP RPKM values. Z-score is calculated from the mean RPKM as z = (Mutant–WT) / SQRT(|Mutant|+|WT|). Volcano, scatter, and box-whisker plots were generated using VisRSeq (version 0.9.12). Genome browser snapshot were captured using IGV (version 2.4.3). Volcano, scatter, and box-whisker plots were generated using VisRSeq (version 0.9.12). Genome browser snapshot were captured using IGV (version 2.4.3).

Strand-specific RNA-seq were aligned using STAR aligner (version 2.5.3a, default parameters) to transcriptome of hg19. Reads with identical start and end coordinates were discarded as PCR duplicates. Reads were then filtered for mapping quality of > 5. Gene expression RPKM were calculated using only strand-specific reads using SeqMonk using the ENSEMBL gene annotation. Triplicate RNA-seq were averaged prior to calculation of z-score. Z-score is calculated from the mean RPKM as z = (Mutant–WT) / SQRT(Mutant+WT). P-value is generated from 2-tailed t-test. Significant deregulation threshold for RNA-seq: |z| > 1.5 or 0.8, p-value < 0.05. Significantly enriched Gene Ontology were called using PANTHER.

Flow Cytometry

Plots

Confirm that:

☒ The axis labels state the marker and fluorochrome used (e.g. CD4-FITC).

☒ The axis scales are clearly visible. Include numbers along axes only for bottom left plot of group (a 'group' is an analysis of identical markers).

☒ All plots are contour plots with outliers or pseudocolor plots.

☒ A numerical value for number of cells or percentage (with statistics) is provided.

Methodology

Sample preparation	Samples were resuspended in 2% CCS in PBS and stained with indicated antibodies for 45 min. Cell were then washed with 2% CCS prior to running flow cytometry.
Instrument	BD LSRFortessa (model #647794L6)<br>BD FACSAria Fusion
Software	DIVA version 8.0 (build 2013 07 02 02 11); Firmware version 1.4 (BD LSRFortessa); CST version 3.0; PLA version 2.0<br>FlowJo version x 10.0.7r2 and 10.1r5
Cell population abundance	Flow cytometry was performed after sort to confirm purity >99%.
Gating strategy	Gating strategies were based on previously published work. (van Galen et al. Cell stem cell, 2014; Laurenti et al. Cell stem cell, 2015; Notta et al. Science, 2016; Notta et al. Science, 2011; Doulatov et al. Nature immunology, 2010)

☒ Tick this box to confirm that a figure exemplifying the gating strategy is provided in the Supplementary Information.
